# Emerging Roles of Filopodia and Dendritic Spines in Motoneuron Plasticity during Development and Disease

**DOI:** 10.1155/2016/3423267

**Published:** 2015-12-30

**Authors:** Refik Kanjhan, Peter G. Noakes, Mark C. Bellingham

**Affiliations:** School of Biomedical Sciences, The University of Queensland, Brisbane, QLD 4072, Australia

## Abstract

Motoneurons develop extensive dendritic trees for receiving excitatory and inhibitory synaptic inputs to perform a variety of complex motor tasks. At birth, the somatodendritic domains of mouse hypoglossal and lumbar motoneurons have dense filopodia and spines. Consistent with Vaughn's synaptotropic hypothesis, we propose a developmental unified-hybrid model implicating filopodia in motoneuron spinogenesis/synaptogenesis and dendritic growth and branching critical for circuit formation and synaptic plasticity at embryonic/prenatal/neonatal period. Filopodia density decreases and spine density initially increases until postnatal day 15 (P15) and then decreases by P30. Spine distribution shifts towards the distal dendrites, and spines become shorter (stubby), coinciding with decreases in frequency and increases in amplitude of excitatory postsynaptic currents with maturation. In transgenic mice, either overexpressing the mutated human Cu/Zn-superoxide dismutase (hSOD1^G93A^) gene or deficient in GABAergic/glycinergic synaptic transmission (gephyrin, GAD-67, or VGAT gene knockout), hypoglossal motoneurons develop excitatory glutamatergic synaptic hyperactivity. Functional synaptic hyperactivity is associated with increased dendritic growth, branching, and increased spine and filopodia density, involving actin-based cytoskeletal and structural remodelling. Energy-dependent ionic pumps that maintain intracellular sodium/calcium homeostasis are chronically challenged by activity and selectively overwhelmed by hyperactivity which eventually causes sustained membrane depolarization leading to excitotoxicity, activating microglia to phagocytose degenerating neurons under neuropathological conditions.

## 1. Introduction

It has been well over 100 years since spines on dendrites of cerebellar Purkinje cells of hen were first discovered by Ramon y Cajal in 1888 [1, 2]. Subsequently he identified dendritic spines and filopodia in other neurons including cortical and hippocampal pyramidal cells, cerebellar basket and Golgi cells, and spinal motoneurons from various species including humans, cats, dogs, birds, and rabbits at different developmental stages from embryonic to adulthood [3], using the Golgi method [4]. Cajal noted that spine density was higher in early postnatal development than at later stages. He made the first observation of spine plasticity in pyramidal neurons and proposed that the spines might help to increase and modify synaptic connections. Dendritic spines have fascinated scientists ever since and were assumed to underlie the physical substrate of long-term memory in the brain, after the first electron microscopic analysis of spines in cortical neurons [5]. Despite more than a century of research, a definitive role for dendritic spines remains elusive: a recently emerging view is that they are strategically positioned postsynaptic cellular compartments likely to play key roles in neuronal functions such as information processing and plasticity under normal and neuropathological conditions [6–22].

**Figure 1:**
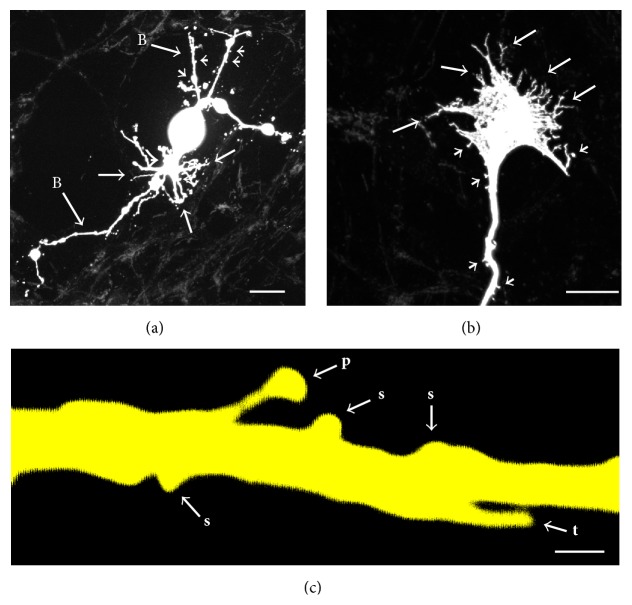
(a and b) show formation of filopodia and spine-like processes (protospines) on neuritis and dendritic growth cones from developing (immature) human cortical neuron-like cells derived from induced pluripotent stem (iPS) cells in culture without synaptic inputs. These cells were dye-filled from their soma with a less invasive semiloose seal Neurobiotin electroporation method, and Neurobiotin was visualized by incubating cells in Streptavidin Cy3 (for methods see Kanjhan and Vaney, 2008 [25]). (a) shows the formation of neurite branches (“B-arrows”), filopodia (long arrows), and spine-like processes or protospines (short arrows) protruding from the soma and neurites in a developing immature neuron-like cell. (b) shows a high-magnification image of a dendritic growth cone with filopodia (thin long processes) and spine-like processes (shorter protrusions; short arrows) protruding from its circumference, in the absence of any synaptic inputs. The same cell had a much longer axonal growth cone extending from soma in opposite direction (not shown). (c) shows the types of spines found in the dendrites of a hypoglossal motoneuron from a 15-postnatal-day-old wild-type C57/Bl6 mouse. In this panel, all types of spines previously reported in other neuronal types are evident. These include (p) pedunculated spines that are thin and longer with prominent necks and heads resembling mushrooms; (s) sessile spines that show stubby or short lacking clear necks; (t) thin spines that are longer filopodia-like spines and lack clear necks and mushroom-like heads. All images were taken with a 100x oil objective (NA 1.35) using 2.5x (b) and 10x (c) optical zoom using an Olympus BX61 (Olympus Fluoview ver. 1.7c) microscope. Each micrograph is a confocal image stack of 10 × 0.35 *μ*m (a and b) and 3 × 0.35 *μ*m (c) thick optical sections. Scale bar = 10 *μ*m in (a-b) and 1 *μ*m in (c).

Dendritic spines are protrusions from the dendritic shaft of neurons (Figure 1) that comprise the receptive postsynaptic compartment at most excitatory synapses in the brain [5, 6, 9, 23]. Time-lapse imaging of dendritic spines in hippocampal slices has revealed an amazingly plastic structure that undergoes continuous changes in shape and size, which are not intuitively related to its assumed role in long-term memory and neuroplasticity [7, 10]. The spine can dynamically form, change its shape, and disappear in response to afferent stimulation, indicating that spine morphology and density are an important vehicle for structuring synaptic interactions and plasticity [7, 9, 10, 12, 17, 20, 22, 24]. Functionally, the spine has been shown to be an independent cellular compartment, able to regulate calcium concentration independently of its parent dendrite [8]. While this role is crucial in the developing nervous system, large variations in spine shape and density in the adult brain and under neuropathological conditions indicate that tuning of synaptic inputs and plasticity may be a role of spines throughout the life of a neuron [9, 11–13, 17, 19, 20, 22].

Most of our knowledge today about dendritic spines and filopodia has primarily come from studies on cortical, hippocampal, and cerebellar neurons. Our recent research suggests significant roles for dendritic spines and filopodia in motoneuron function and plasticity, particularly during embryonic-postnatal development and under neuropathological conditions. In the following sections, we will review previous studies of filopodia and spines in motoneurons in the light of these recent advances.

## 2. Morphological Studies of Filopodia and Spines on Motoneurons: A Historical Perspective

Motoneurons located in the lower brainstem (i.e., cranial motor nuclei III to XII) and through the entire length of the spinal cord play vital roles in the control of motor functions such as respiration, posture, and locomotion. Humans possess more than 500 different skeletal muscles, capable of contracting in a precise temporal and spatial coordination to execute many refined complex motor functions. Motoneurons have developed elaborate dendritic structures to meet these highly complex demands. Somatic and dendritic motoneuron morphology was first revealed in the drawings of Ramon y Cajal using the Golgi method [1, 2]. Growth cones, filopodia, and spines can be seen on dendrites of embryonic chicken and adult cat spinal motoneurons [1–3].

Subsequently a number of studies, primarily using the Golgi method, described the presence of filopodia and spines on motoneurons [28–31]. In their early studies in the adult cat and monkey, M. E. Scheibel and A. B. Scheibel described that the majority of motoneuron dendrites were spine-bearing, but their distribution was not nearly as regular as in cortical neurons [28]. Scheibel and colleagues however in their following studies described spine-like processes as protospines (filopodia-like long thin immature spines with no obvious necks) on the soma and dendrites (proximal and distal), and they argued that these or primitive polymorphic spines were a feature of perinatal motoneuron dendrites, present only during early development with a peak around postnatal day 11 (P11) and then declining in numbers due to resorption onto the dendritic surface or shafts, almost entirely disappearing with formation of dendritic bundles [29, 30, 32]. They suggested that repository spinal programs involved in controlling rhythmic behaviors such as respiration and locomotion were originally loaded via an archaic system of presynaptic fibers terminating on the polymorphic protospines covering most of the dendrites and soma during the prenatal phase and were subsequently lost with rearrangement of dendrites in tightly packed bundles around the fascicles of myelinated axons. The programs were then conceived to function autonomously, as loss of spines coincided with development of bundles, throughout the organism's existence, subject to modification and override by newer systems [29, 30, 32]. These conclusions of M. E. Scheibel and A. B. Scheibel resulted in a loss of interest in investigation of dendritic spines on motoneurons by many laboratories around the world. A significant number of subsequent studies, some of which are mentioned above, using various methods, including Golgi, retrograde tracing, calcium imaging, and dye-filling, did not report or discuss filopodia or spine presence on motoneurons in the adult or during development [33–37]. This will be discussed in detail later in this section (see below).

Despite this setback emanating from M. E. Scheibel and A. B. Scheibel's conclusions, at least some Golgi studies consistently reported the presence of spines or filopodia on motoneurons from various species studied. In the rat, subtle differences were noted in the descriptions derived from neonatal and young adult Golgi preparations [38]. For example, in most of the neonatal materials, profuse spine-like excrescences, the heteromorphic protospines were noted on the somatodendritic domains of motoneurons. At P10- to P14-day-old juvenile rats, the protospines became fewer and were comparable numerically to the spines counted on motoneurons of much older preparations (35 to 65 days postnatally) [38]. Interestingly, this loss of protospines coincides with the refinement of motor neuron circuit, namely, the loss of polyneuronal innervation of muscle [39–41]. Following this developmental period, occasional sessile (stubby and short spines lacking clear necks) and rare pedunculated (thin and longer spines with prominent necks and heads resembling mushrooms) spines and appendages on the soma and dendrites of hypoglossal motor neurons from adult primates have been reported [42].

Development of electron microscopy techniques in the 1950s revealed morphological and synaptic properties of spines at ultrastructural level [5]. In the 1970s and 1980s Vaughn put forward his synaptotropic hypothesis on spine and synapse formation based on his ultrastructural and Golgi studies on embryonic and newborn mouse spinal cord motoneurons, where he reported synaptic contacts occurring on motoneurons as early as embryonic day 11 (E11) [43–46]. Vaughn's synaptotropic hypothesis postulates that dendritic filopodia, arising from growth cones capture axons, establish synaptic contacts and then gradually turn into spines or dendritic shafts and that filopodia also produce motoneuron dendritic branches [3, 12, 45–47]. This filopodial model of spine, synapse, and dendrite formation will be discussed in detail below in Section 4. Other scientists have also studied synaptic density and contacts at ultrastructural level in various motoneuron pools in adult animals, including rat phrenic [48] and hypoglossal [49] and cat lumbosacral motoneurons [50].

In the 1980s, development of neuroanatomical and retrograde tracing of motoneurons from their target muscles with wheat germ agglutinin (WGA) or cholera toxin- (CT-) conjugated horseradish peroxidase (HRP) and biocytin and fluorescent tracers (e.g., Fluorogold) provided a new tool targeting of specific motoneuron populations or pools in different species [31, 51–55]. However, most of these studies like those indicated before barely mentioned dendritic spines or filopodia in motoneurons.

Another major development in the study of motoneurons started in the early 1950s, with intracellular microelectrode recordings from spinal motoneurons allowing a detailed analysis of their electrophysiological properties [56]. Use of sharp electrodes also allowed the development of dye-filling methods of individual motoneurons in the 1960s [57]. The morphology of functionally characterized different types of mammalian spinal and brainstem motoneurons started to emerge from this type of* in vivo* research [58, 59]. Subsequent studies, using dye-filling with HRP, fluorescent dyes (e.g., Lucifer Yellow), and biocytin or Neurobiotin revealed dendritic morphology of functionally characterized motoneurons primarily from the* in vivo* adult cat studies [60–65], followed by studies in the adult rat* in vivo* [66–69] and in developing rat brainstem slices [70, 71].

Dye-filling studies of individual motoneurons reported occasional sessile and rare pedunculated spines and appendages on the soma and dendrites of motoneurons from adult animals including cat hypoglossal [65], phrenic [72], and hind limb [62] motoneurons and rat phrenic motoneurons [31]. A developmental study identified frequent growth cones, filopodia, and lamellipodial and fusiform processes in kitten at birth, most processes disappearing at P45 [62]. Therefore, one possibility is that, in older animals, the spines or spine-like processes (e.g., filopodia or appendages) are significantly reduced compared to newborns; however, another study reported a few spines in developing cat phrenic motoneurons [73], with a similar spine density to that of adult cat phrenic motoneurons [72]. By contrast, another dye-filling study in developing rat (P1 to P30) hypoglossal motoneurons, despite performing detailed morphometric analysis, did not mention anything about dendritic spines or filopodia [70, 71].

In the past 15 years mice have increasingly become the species of choice for studying mammalian motoneurons, primarily due to the availability of transgenic mice. In mice, most of the available information about motoneuron dendritic structure comes from studies that used retrograde tracers and immunochemical or Golgi staining to visualize and draw projections of motoneuron dendrites in single sections without full reconstruction [74–78]. The quantification of motoneuron dendritic spines by Golgi or HRP staining combined with light microscopy is limited due to dense and dark reaction products, as significant number of spines under or above the dendrites are likely to be unaccounted for [79]. Also with these dark reaction products, it would be hard to detect some of the very short and stubby spines (0.1 to 0.4 *μ*m long), as shown in Figure 1(c) at high magnification. Although some of these difficulties have been overcome recently by using fluorescent molecules such as Fluorogold in retrograde labeling studies [80] or using transgenic mice with fluorescently (e.g., Green Fluorescent Protein (GFP) or Yellow Fluorescent Protein (YFP)) expressing motoneurons [81], there are still potential limitations with nonspecific and partial uptake of labels not reaching intensities sufficient to reveal fine structural detail such as dendritic spines in distal dendrites (see below) [55]. Only a handful of studies have carried out dye-filling studies of individual motoneurons in mice using* in vitro* hemisected lumbar spinal cords from P3 to P13 mice [82] and in brainstem spinal cord preparations from P3 to P9 mice [83, 84]. Dendritic elongation and branching abnormalities of lumbar motoneurons were reported in mutated human Cu/Zn-superoxide dismutase (hSOD1^G85R^) overexpressing mice compared to wild-type mice at P3–P9 [83, 84]. It is again surprising that there was no mention of spines and filopodia on neonatal mice lumbar motoneurons dye-filled with biocytin or Neurobiotin [82–84] or Ca^2+^ Green-1 and Texas Red dextrans [37]. Of these earlier studies in the mice, only one study reported and quantified dendritic spines in cervical motoneurons using a rapid Golgi method [74]. This study reported significant reduction in dendritic length, branching, and spine density in presumed alpha motoneurons from the 2–6-month-old* Wobbler* mouse (a model of human infantile spinal muscular atrophy) compared to wild-type mice [74].

**Figure 2:**
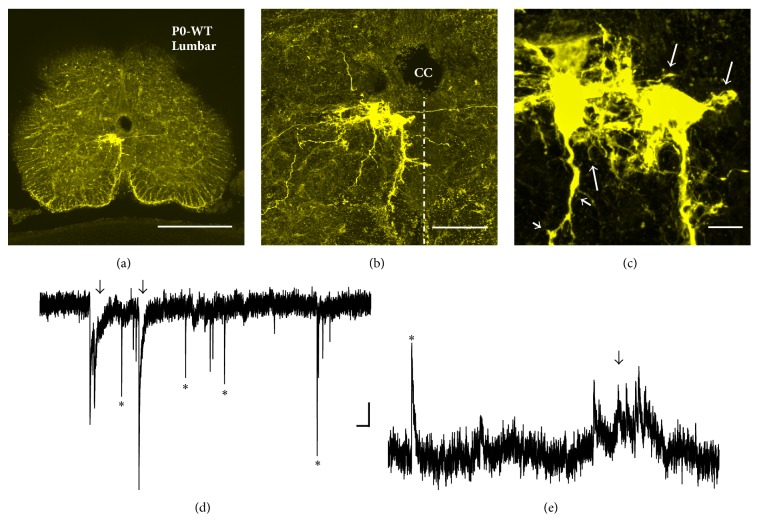
The somatodendritic and synaptic properties of lumbar motoneurons from a newborn wild-type C57/Bl6 (P0-WT) mouse. (a) Low-power image showing a lumbar spinal cord slice in transverse plane and the location of dye-filled motoneurons (yellow cells). (b) Medium-power confocal image showing two dye-coupled motoneurons located ventrolateral to the central canal (CC), with commissural dendrites crossing the midline (dashed line) to the contralateral side of the spinal cord. (c) High-power confocal image of these two motoneurons, which displayed action potential firing upon membrane depolarization (not shown), each displaying extensive filopodia (long arrows) and spine-like processes (short arrows) present in their somatodendritic domains. (d) Excitatory postsynaptic currents (downward deflections) recorded at a holding potential of −60 mV. Fast inactivating excitatory currents may be AMPA receptor-mediated (asterisks), whereas slowly inactivating excitatory currents likely include NMDA currents alone or together with AMPA (short arrows). (e) Inhibitory postsynaptic currents (upward deflections) recorded at a holding potential of 0 mV. Fast inactivating inhibitory currents may be glycine-mediated (asterisk), whereas slowly inactivating currents likely include GABA-mediated currents (short arrows). Scale bar is 200 *μ*m in (a), 50 *μ*m in (b), and 10 *μ*m in (c). Scale bars for (d and e) are 250 ms and 25 pA.

**Figure 3:**
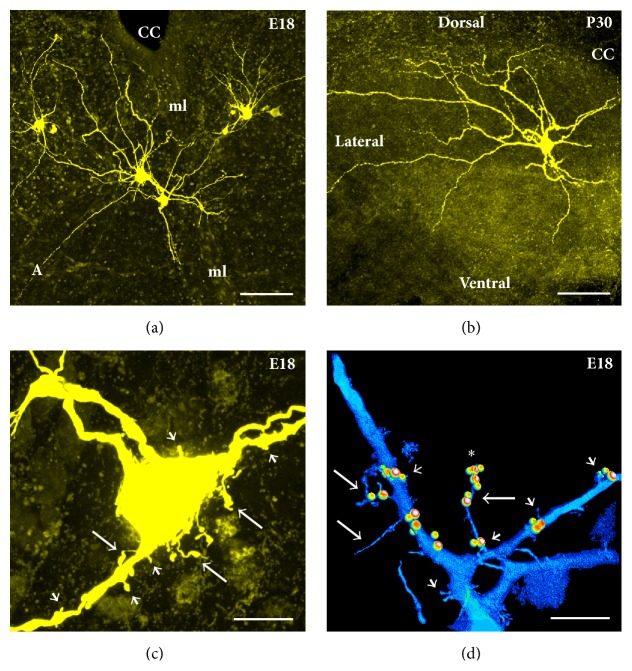
Morphological properties of hypoglossal motoneurons in brainstem slices obtained from embryonic and adult C57/Bl6 wild-type mice (for methodology see Kanjhan and Bellingham, 2013 [26, 27]). (a) Image showing 4 hypoglossal motoneurons filled with Neurobiotin in a 300 *μ*m slice preparation obtained from a mice at embryonic day 18 (E18). Note that the motoneuron on the dorsal right-hand side is dye-coupled to 4 adjacent motoneurons. Two motoneurons on the ventromedial portion of the hypoglossal nucleus have dendrites crossing the midline (ml) to the contralateral side. Axon (A) of one of the motoneurons is clearly visible projecting in the ventrolateral direction to join the hypoglossal nerve outlet. (b) A hypoglossal motoneuron from an adult mouse at postnatal day 30 (P30). Note a significantly larger dendritic tree in the adult mice. (c) A high-power confocal image showing filopodia (long arrows) and spine-like processes (short arrows) at the soma and primary dendrites of a motoneuron from a WT mouse at E18. (d) A rendered 3D reconstruction generated by Imaris software illustrating an overlapping localization of the presynaptic vesicular glutamate transporter-2 (VGLUT-2) terminals (small spheres) and the postsynaptic density protein-95 (PSD-95) (larger spheres) on filopodia (note as many as 4 excitatory synaptic contacts on a single filopodium marked as *∗*) and spine-like processes on the primary dendrites of a motoneuron from an E18 WT mouse. CC: central canal. Scale bars = 100 *μ*m in (a and b); 10 *μ*m in (c and d).

Recently, we have developed a highly sensitive and less invasive (i.e., minimal cellular damage) dye electroporation method to fill individual cells using Neurobiotin, a small molecule (molecular weight (MW) = 323) with the advantage of comprehensive intracellular distribution (Figures 1, 2, and 3) [25, 26, 85]. We have been using voltage pulses to electroporate the membrane in giga-seal or semiloose seal modes, instead of direct and variable suction that can damage the plasma membrane and intracellular organelles [25, 26, 85]. Combining our less-invasive and sensitive Neurobiotin electroporation method with the high magnification (100x objective with 2.5x to 10x optical zoom) laser confocal and super resolution microscopy has allowed detection and quantification of significant numbers of dendritic spines and filopodia in developing hypoglossal motoneurons from mouse brainstem slices (Figures 1(c) and 3) [26, 27]. Using this method we have recently studied somatodendritic morphology of over 100 hypoglossal motoneurons in developing mice from E17 to P28 [86]. Our most recent studies on 300–500 *μ*m transverse spinal cord slice preparations from newborn mice also indicate the abundant presence of filopodia and spines on somatodendritic domains of dye-filled lumbar motoneurons (Figures 2(a)–2(c)) that received both excitatory glutamatergic NMDA (*N*-methyl-D-aspartic acid) and AMPA (*α*-amino-3-hydroxy-5-methyl-4-isoxazolepropionic acid) and inhibitory gamma-aminobutyric acid (GABA) and glycinergic synaptic currents, based on their activation/inactivation kinetics (Figures 2(d)-2(e)). In motoneurons we find all types of spines previously reported in other neuronal types [6, 12, 23], including pedunculated (thin and longer spines with prominent necks and heads resembling mushrooms), sessile (stubby or short spines lacking clear necks), and thin and longer filopodia-like spines lacking clear necks (Figure 1(c)). We have also occasionally seen branched spines with Y-shaped tips in dendrites of developing motoneurons (see Figure 6(c), asterisk). General understanding is that mushroom (pedunculated) and stubby (sessile) spines represent more mature and stable spines, while thin and branched spines tend to be more plastic and immature [6, 12, 23]. In the following sections, we will show examples and argue that the filopodia and spine-like structures form the structural bases of motoneuron plasticity during development and under neuropathological conditions.

Reasons for lack of detection or reporting of spines and filopodia on motoneuronal somatodendritic domain by many previous morphological studies are not clear. We believe there may be many contributing factors such as significant neuronal injury and tissue damage during recording or dye-filling, sensitivity of detection method and dye used, and sufficient magnification (minimum 100x objective with 2.5x optical zoom) to detect short and stubby spines (0.1 to 0.4 *μ*m long; Figure 1(c)) that are the most common type in the adult. We noted that most previous morphological studies mentioned above, especially prior to use of confocal microscopy, did not image cells at high enough magnification to identify spines and filopodia on motoneurons. From our experience, any major physical damage to the cellular soma or dendrites during whole-cell patch-clamping, electrode pull-out, prolonged recordings with patch or sharp (high impedance intracellular recordings* in vivo*) electrodes under unstable conditions (i.e., movement), or using inappropriate pipette solutions (e.g., osmolarity, ionic composition, and pH), can result in membrane damage and subsequent swelling, vacuolization, blebbing, or beading of cellular membranes or compartments [25, 26, 85]. Under such conditions, either filopodia and spines will become undetectable due to retraction or they will simply integrate with swellings, vacuoles, blebs, or beads and disguise upon losing their structural support. Indeed when we closely look at the most of the previous morphological studies (see above), especially in* in vivo* animal preparations with intracellular filling including our own work [68, 69], beading and blebbing are common in somatodendritic domains of motoneurons. Dendritic spines can also structurally restrict access of dyes, especially larger molecules such as HRP (MW ~ 44 kilo Dalton (kDa)) compared to small size Neurobiotin (MW = 323) (Kanjhan, unpublished observations). Furthermore, the intensity of dye is always much higher in dendritic shafts, particularly in distal dendrites, compared to spines protruding from them, even with smaller dyes Neurobiotin, biocytin (MW = 372), Lucifer Yellow (MW = 522), and Alexa Fluor (MW = 570) [25]. Another limitation is the working distance of the objectives and penetration of light through the 300 *μ*m thick brain tissue; the spines and filopodia can reliably be detected and imaged only if they are located on the surface of the tissue (ideally within the top 20 *μ*m). The clarity and resolution of the image are lost in dendrites deeper than 25–30 *μ*m from the surface of the brain slice and the spines become too blurry for any reliable detection and measurement. One way around this problem could be further slicing of the 300 *μ*m thick brain slice to ≤20 *μ*m sections following fixation of the tissue.

## 3. Molecular Mechanisms Involved in the Development of Motoneuron Dendritic Trees, Filopodia, and Spines: Remodeling under Neuropathological Conditions

During early embryonic development, motoneurons emerge from dividing progenitor cells located in the medial portion of the ventral neural tube [87]. Motoneuron identities are established by patterning cues working in cooperation with intrinsic sets of transcription factors [87–92]. As the embryo develops, motoneurons further differentiate in a stepwise manner to form compact anatomical groups, termed motor pools or motor columns, connecting to a unique muscle target [88, 92–94]. The lateral motor column (LMC), positioned in the brachial and lumbar enlargements of the spinal cord, contains motoneurons that innervate the skeletal muscles of the limbs, while the mediomedial column (MMC), positioned throughout the rostrocaudal extent of the spinal cord, contains motoneurons that for the most part innervate the axial trunk muscles [92–94].

Motoneurons are unique in the vertebrate central nervous system, in the sense that they are arguably the only neurons for which both function and precise target tissue innervation are known. Since the pioneering work of Sir Charles Scott Sherrington, it is widely recognized that motoneurons link the central nervous system to the muscles [95]. Motoneurons are thus the final common effector pathway, where all the peripheral sensory and central premotor and interneuron pathways converge to elicit motor output. A single motoneuron drives a subset of muscle fibers within a muscle, forming a motor unit [95].

Motoneuron pools are not homogeneous and comprise diverse subtypes, according to the muscle fiber type they innervate [41, 89, 96, 97]. Based on their somatodendritic structure, synaptic inputs, axonal projection, and gene expression profiles, every motor column consists of three major motoneuron subtypes: the alpha, beta, and gamma motoneurons; with further subdivision, seven categories of motoneurons have been described based on their innervation pattern [36, 89, 96, 97]. The large multipolar alpha motoneurons innervate the extrafusal muscle fibers in the skeletal muscle and receive input from the proprioceptive sensory afferent neurons. Alpha motoneurons form three types of motor units: (1) fast-twitch fatigable (FF) alpha motoneurons have the biggest soma size and axon diameter, control a large number of type IIB extrafusal muscle fibers, have large neuromuscular synapses, and display phasic/delayed firing patterns; (2) fast-twitch fatigue-resistant (FR) alpha motoneurons are slightly smaller than FF motoneurons, innervate type IIA extrafusal muscle fibers, have large neuromuscular synapses, and display high-frequency tonic/delayed firing patterns; (3) by contrast, slow-twitch (S) alpha motoneurons are smaller than FF and FR types, innervate fewer type I myosin heavy chain (MHC) muscle fibers, form smaller neuromuscular synapses, and produce tonic/immediate action potential firing [36, 89, 96, 97]. The morphological properties of beta motoneurons resemble alpha motoneurons, but they are skeleton-fusimotor and innervate both the extrafusal fibers in the skeletal muscle and the intrafusal muscle fibers in the muscle spindle. There are two types of beta motoneurons: (1) beta static motoneurons innervate type IIA or IIB extrafusal fibers and the intrafusal nuclear bag2 fiber; (2) beta dynamic motoneurons innervate type I extrafusal muscle fibers and the intrafusal nuclear bag1 fiber [89, 96, 97]. We currently do not know much about specific properties of the beta motoneurons. The gamma motoneurons, which make up for ~30% of all motoneurons, are the smallest of all motoneuron types and innervate exclusively the intrafusal muscle fibers in the muscle spindles, without a direct input from proprioceptive afferents. There are two types of gamma motoneurons: (1) gamma static motoneurons innervate the intrafusal nuclear bag2 fiber and/or the nuclear chain fibers; (2) gamma dynamic motoneurons innervate the intrafusal nuclear bag1 fiber [89, 96, 97]. The gamma motoneurons can also be distinguished from the neuronal nuclear antigen (NeuN) expressing alpha motoneurons, on the basis of expression of transcription factor Err3 (estrogen receptor-related protein 3) or a muscle spindle-derived signaling molecule Wnt-7a (wingless type 7a) [36, 89, 90].

Motoneurons are cholinergic neurons that receive excitatory glutamatergic and inhibitory GABAergic and glycinergic synaptic inputs, as well as many additional modulatory inputs including noradrenergic, serotonergic, dopaminergic, cholinergic, and purinergic inputs [33–35, 97–102]. The intrinsic membrane properties of motoneurons are precisely tuned within each category of motoneurons in order to produce an output that is adapted to the contractile properties of their specific muscle targets [97, 103]. The voltage-dependent delayed rectifier K^+^ channel (Kv2.1) contributing to neuronal excitability has been specifically located at the postsynaptic site of large cholinergic C-bouton inputs to somatodendritic domains of alpha motoneurons [104]. There is some evidence that primarily large alpha motoneurons, especially FF type phasic motor units, are selectively lost in aging and motoneuron diseases; their neuromuscular junctions (NMJs) become first denervated (i.e., endplate denervation) and are then partially reinnervated or compensated by the newly grown axon collaterals of adjacent motoneurons [41, 89, 105–107]. Alpha motor axon terminals at NMJs have been shown to undergo bouts of degeneration and regeneration in young asymptomatic SOD1^G93A^ mice, but later in life alpha motor axons selectively degenerate via a process termed “dying back,” resulting in the appearance of neurological symptoms due to denervation of muscle fibers and loss of motor neurons [41, 107, 108].

Intercellular communication is essential for the regulation of embryonic development. During early development at the time of cell division, immature neurons start to put out processes known as growth cones that bear filopodia, spine-like structures, and small branches, as shown here with dye-filling by Neurobiotin electroporation of developing human cortical neurons (equivalent of less than 90-day-old fetal neurons) derived from induced pluripotent stem cells (Figures 1(a)-1(b)). Note that isolated developing fetal cells in culture conditions are able to form filopodia and spines from their growth cones in the absence of synaptic inputs (Figures 1(a)-1(b)). Growth cones and filopodia on motoneurons were first described by Ramon y Cajal [1–3]. The growth cones give rise to filopodia, which are composed largely of filamentous- (F-) actin bundles or polymers [47, 109]. Filopodia are long (~2–20 *μ*m) and thin (<0.3 *μ*m in diameter) protrusions or stalks that lack a knobby head (Figures 1(a), 2(c), and 3(c)-3(d)); they are present during development on the soma, dendrites, and axons of neurons and are much more dynamic than dendritic spines [3, 12, 47, 110–112]. It is rare to see filopodia on mature neurons, and therefore their function may be primarily developmental, except under pathological conditions when they may reappear as a regenerative response to injury [62, 111–114]. However, a number of manipulations have also been shown to induce filopodia growth including high-frequency focal synaptic stimulation by activation of glutamatergic NMDA receptors, overexpression of glutamatergic AMPA receptor subtype 2 (GluR2) and the transmembrane agrin (TM-agrin), proteolytic cleavage of agrin by neurotrypsin, or activation of the small conductance Ca^2+^-activated K^+^ channel subtype 3 (SK3) [3, 12, 47, 110].

Activation of the central regulators of actin dynamics Ras (rat sarcoma) and Ras homolog (Rho) family of small GTPases (guanosine triphosphatases), including most studied members RhoA, Rac1 (Ras-related C3 botulinum toxin substrate 1), and cdc42 (cell division cycle 42), and their downstream intermediates results in the polymerization of actin fibers by enabled vasodilator-stimulated phosphoprotein (Ena/Vasp) homology proteins [112, 115]. Growth factors bind to receptor tyrosine kinases resulting in the polymerization of actin filaments, which, when cross-linked, make up the supporting cytoskeletal elements of filopodia [112, 115–118]. Rho activity also results in activation by phosphorylation of ezrin-radixin-moesin (ERM) family proteins that link actin filaments to the filopodia membrane [115]. Myosin-X (Myo10) is a MyTH4-FERM (myosin tail homology 4-for protein 4.1, ezrin, radixin, and moesin) myosin that is a molecular motor localized to the tips of filopodia and functions in filopodia formation by acting downstream of small GTPase cdc42 [119]. Filopodia are much more dynamic than dendritic spines, and issues that are not understood or clear include the role of Ca^2+^ influx in filopodia dynamics [112].

**Figure 4:**
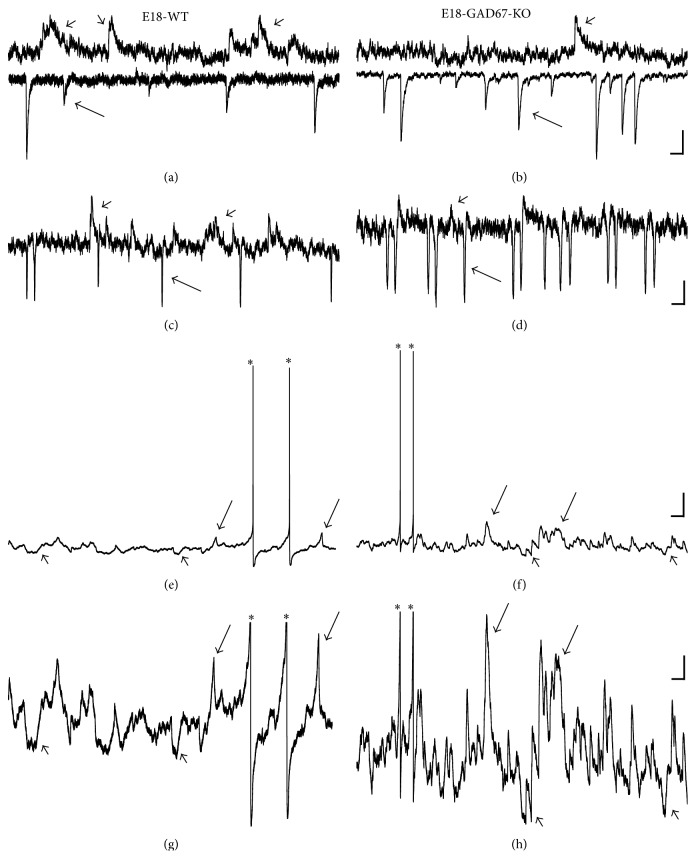
Electrophysiological recordings of hypoglossal motoneurons from C57/Bl6 WT mice compared to GAD67-KO mice at E18 (for methodology see Kanjhan and Bellingham, 2013 [26]). (a-b) Somatic recordings of excitatory (downward deflections, long arrows, EPSCs) and inhibitory (upward deflections, short arrows, IPSCs) postsynaptic currents using low resistance electrodes (3-4 mΩ) at −60 mV and 0 mV holding potentials, respectively. (c-d) Dendritic recording of EPSCs (downward deflections, long arrows) and IPSCs (upward deflections, short arrows) at 0 mV holding potential using higher impedance electrodes (10–15 mΩ). (e-f) Current-clamp recording of membrane potential at action potential (*∗*) firing threshold, showing subthreshold excitatory (long arrows, EPSPs) and inhibitory (short arrows, IPSPs) postsynaptic potentials. (g-h) Magnified baseline traces of (e)-(f), respectively. Scale bars = (a-b) = 50 pA, 0.1 s; (c-d) = 25 pA, 0.1 s; (e-f) = 20 mV, 1 s; and (g-h) = 3 mV, 1 s.

Rats and mice are born with relatively immature forebrains, and spinogenesis in cortical pyramidal neurons starts postnatally; at this stage these neurons are primarily depolarized by GABA instead of glutamate, as excitatory glutamatergic synapses are not formed yet [120]. By contrast, motoneurons and especially hypoglossal motoneurons, which need to be functional at birth for the newborn to breath and suckle, reach maturity at late embryonic stages (Figures 3 and 4). Our results show abundant filopodia and spine-like processes at E17-E18 motoneurons, some of which contain both pre- and postsynaptic components of glutamatergic synapses: vesicular glutamate transporter 2 (VGLUT2) and postsynaptic density 95 (PSD-95), respectively (Figures 3(c)-3(d)). Some of the long filopodia had more than one VGLUT2 and PSD-95 appositions (Figure 3(d)), suggesting multiple potential synaptic sites. These observations are in agreement with Vaughn's ultrastructural findings that synaptic contacts form in mice spinal motoneurons primarily on filopodia as early as E11-E12 [44]. The spinal cord starts to convey first synaptic activity recorded from motoneurons at E12.5, that is, GABAergic [102]. GABAergic and cholinergic signaling together generate earliest spontaneous spinal motor activity [102]. GABAergic synaptic transmission to motoneurons is soon supported by glycinergic synaptic transmission. Glutamatergic synaptic transmission to motoneurons likely activates at around E14.5 [102]. Functionality of excitatory and inhibitory synapses at E18 has been confirmed by our electrophysiological recordings from motoneurons, which were subsequently filled by Neurobiotin electroporation for morphological analysis (for methodological details see Kanjhan and Vaney, 2008, and Kanjhan and Bellingham, 2013 [25, 26]). Somatic patch-clamp recordings show that at E18 wild-type (WT) mice hypoglossal motoneurons receive both glutamatergic excitatory postsynaptic currents (EPSCs) and GABAergic/glycinergic inhibitory postsynaptic currents (IPSCs) under voltage clamp (Figure 4(a)). EPSCs and IPSCs are translated into excitatory postsynaptic potentials (EPSPs) and inhibitory postsynaptic potentials (IPSPs) under current-clamp conditions, respectively (Figures 4(e) and 4(g)). In lumbar motoneurons, the earliest age we have tested was P0, and at that age group we have seen well-developed excitatory and inhibitory synaptic currents (Figures 2(d)-2(e)). Activation and inactivation kinetics of these currents suggest presence of fast-inactivating AMPA and slow-inactivating NMDA receptor type-mediated excitatory synaptic currents and fast inactivating glycinergic and slow inactivating GABA-mediated inhibitory synaptic currents (Figures 2(d)-2(e)). We have also studied excitatory and inhibitory synaptic currents in transgenic mice deficient in GABAergic inhibitory synaptic transmission, due to elimination of 67 kDa glutamic acid decarboxylase (GAD-67) enzyme, which is the major isoform catalyzing the decarboxylation of glutamate to GABA in prenatal and neonatal brains. Inhibitory postsynaptic currents are mostly lost in mice that lack GAD-67, while there is an increase in the frequency of excitatory synaptic inputs (Figure 4(b)). Patch-clamp recordings from the proximal dendrites of motoneurons in WT and GAD-67 knockout (KO) mice confirmed these results (Figures 4(c)-4(d)). In addition, dendritic recordings show that at least some of the inhibitory outward currents are generated in the dendrites. While somatic and perisomatic inhibition of motoneurons has been studied in detail [33–35, 121] and immunolabelling studies suggest the presence of dendritic inhibition [122], there is no functional data showing dendritic inhibition in motoneurons [123]. We have recently shown that presynaptic vesicular inhibitory amino acid transporter (VGAT) terminals and postsynaptic GABA_A_ receptor alpha-1 subunits form close appositions on dendrites of hypoglossal motoneurons [27]. Our functional and morphological evidences suggest that inhibitory synaptic modulation may be involved in dendritic integration of synaptic inputs and localized regulation of neuronal Ca^2+^ signaling [124] in motoneuronal information processing [33, 100, 101], as shown in detail for other neuronal types such as retinal ganglion cells [125] and hippocampal neurons [126]. In current-clamp recordings, IPSPs were significantly reduced in amplitude and frequency in hypoglossal motoneurons from the mice deficient in inhibitory synaptic transmission; instead an increase in the frequency and amplitude of EPSPs was observed, which increased the action potential firing probability and reduced the interspike interval (Figures 4(f) and 4(h)). GABA and glycine can activate depolarizing chloride currents in some neurons, such as hippocampal pyramidal cells, during postnatal development due to high intracellular chloride concentrations [102, 120]. Therefore, the presence of IPSCs and IPSPs seen at E18 hypoglossal motoneurons confirms the maturity of hypoglossal motoneurons, as suggested by previous studies [34, 127]. However, the maturation of GABAergic/glycinergic responses from depolarizing to hyperpolarizing may differ among various motoneurons pools [102, 127].

**Figure 5:**
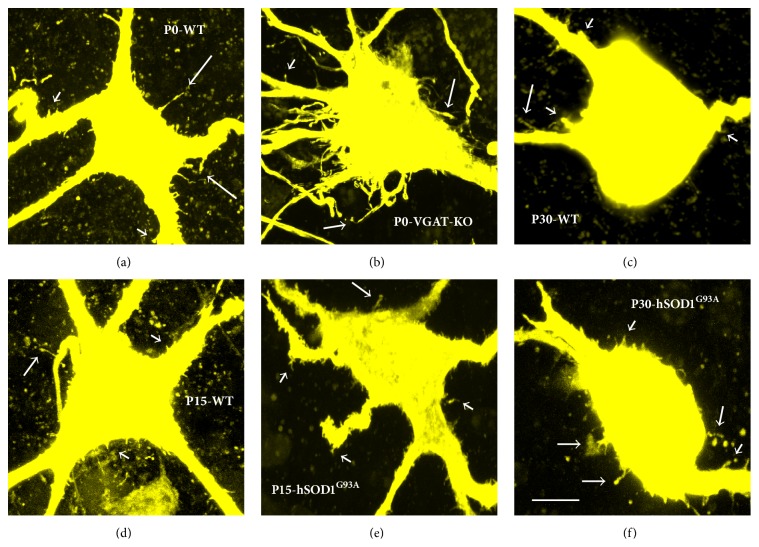
Somatodendritic morphologies of hypoglossal motoneurons during postnatal development and under neuropathological conditions. (a) Filopodia (long arrows) and spine-like processes (short arrows) in the somatodendritic domain of a motoneuron from a P0 WT mouse. (b) Increased density and size of filopodia and spine-like processes from a P0 mice lacking vesicular inhibitory amino acid transporter (VGAT-KO). (c-d) Filopodia and spine-like processes in the soma decrease in density and size during postnatal development from WT mice at P15 (d) and P30 (c). (e-f) Density and size of filopodia and spine-like processes are higher in motoneuronal somatodendritic domain in mice overexpressing the mutated human Cu/Zn-superoxide dismutase (hSOD1^G93A^) gene at P15 (e) and P30 (f). All micrographs are assembled from confocal image stacks of 20 to 40 optical images collected at 0.35 *μ*m steps using an Olympus BX61 (Olympus Fluoview ver. 1.7c) microscope. Scale bar in (f) = 10 *μ*m (applies to all panels).

**Figure 6:**
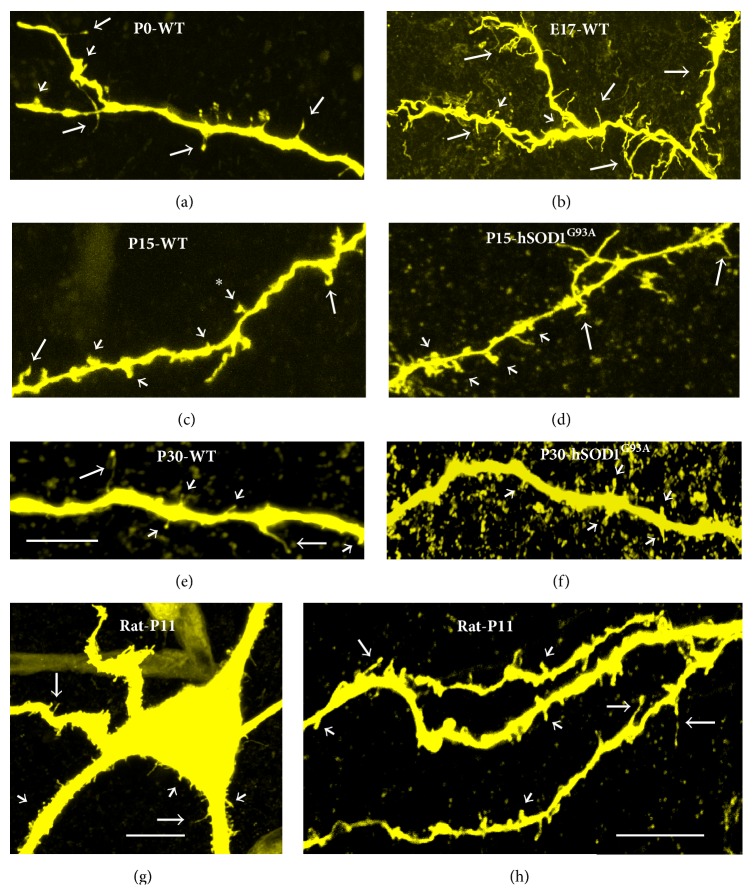
Developmental changes seen in filopodia (long arrows) and spine-like processes (short arrows) in distal dendrites of hypoglossal motoneurons from C57/Bl6 WT mice, Wistar rat (P11; WT), and hSOD1^G93A^ mutant mice. (a-b) The filopodia were long and common and spine-like processes started to form along the distal dendrites at E17-P0. Long and very dense filopodia were seen in some distal dendrites at late embryonic and newborn mice (b). (c, e) Filopodia density compared to E17/P0 gradually reduced with postnatal maturation at P15 (c) and further reduced by P30 (e) WT mice. Spine-like processes decreased in length becoming gradually stubby shaped with postnatal maturation; spine density increased by P15 (c) but decreased at P30 (e). A rare Y-shaped branched spine is marked with an asterisk *∗* (c). (d–f) Increased density of spine-like processes in mice overexpressing the mutated human SOD1^G93A^ gene at P15 (d) and P30 (f). (g-h) Similar filopodia and spine distribution were seen in the P11 rat hypoglossal motoneuronal soma, primary (g) and distal dendrites (h). All micrographs are assembled from confocal image stacks of 20 to 40 optical images collected at 0.35 *μ*m steps. Scale bars in (e), (g), (h) = 10 *μ*m. Scale bar in (e) applies to panels (a) to (f).

**Figure 7:**
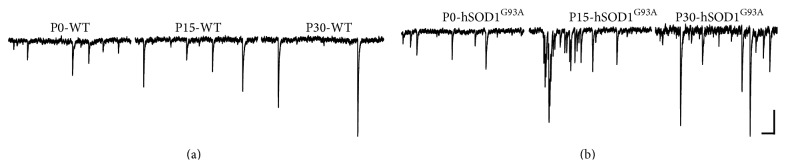
Developmental changes in the amplitude and frequency of spontaneous excitatory postsynaptic currents (EPSCs; downward deflections) in hypoglossal motoneurons recorded from the C57/Bl6 WT mice (a), compared to the mice overexpressing the mutated human SOD1^G93A^ gene (b) at P0, P15, and P30. The amplitude of EPSCs increased by ~3-fold, while the frequency of EPSCs reduced by ~50% with postnatal maturation (from P0 to P30) in WT mice. In the mutated hSOD1^G93A^ mice the frequency of EPSCs was increased, without a clear change in EPSC amplitude, compared to age-matched WT littermates. Scale bars = 100 pA, 0.1 s.

We see filopodia and spine-like processes on the soma and dendrites of developing lumbar (Figures 2(b)-2(c)) and hypoglossal motoneurons from mice (Figures 3(c)-3(d), 5(a), 5(c), 5(d), 6(a)–6(c), and 6(e)) and rats (Figures 6(g)-6(h)). These findings show that the localization of filopodia and spines on motoneurons is not unique to a species or to a specific motoneuron pool. Filopodia-like long processes were also frequently observed on developing motoneuron axons projecting ventrolaterally (not shown). We often saw a variation in density, size, and shape of filopodia and spine-like structures among neighboring motoneurons and even between the dendrites originating from the same motoneuron. In some motoneurons, dorsolaterally projecting dendrites, that likely receive various sensory afferent inputs, had more filopodia and spine-like processes compared to the ventral dendrites. Such specific spine distribution may be important in sensory experience-dependent plasticity of large F type alpha motoneurons that receive sensory inputs. This may have a potential involvement in the selective degeneration of F-type motoneurons in amyotrophic lateral sclerosis (ALS) or motoneuron disease, as well as in aging [41, 89, 105–107]. In the spinal cord, nerve endings from Ia/II proprioceptive sensory neurons, directly contacting alpha motoneurons, are preferentially affected in ALS and degenerate much earlier than those from Ib sensory neurons in SOD1^G93A^ and transactivation response element (TAR) deoxyribonucleic acid- (DNA-) binding protein-43 (TDP-43^A315T^) mutant mice [107]. It is possible that the sensory input changes to motoneurons, such as increases in excitatory synaptic inputs, may be at least partly involved in increases in frequency of excitatory synaptic inputs shown in Figure 7. On motoneuron dendrites we observed both terminal type (at the tips of distal dendrites; Figures 6(a)-6(b)) and collateral type (emerging from dendritic shafts; Figures 3(d), 6(a)–6(c), and 6(h)) filopodia. While the terminal filopodia may be involved in dendritic growth and branching, the collateral filopodia may be involved in spinogenesis or synaptogenesis, as suggested previously [47, 62, 128–130]. Spines on Purkinje and pyramidal cells show morphological variability and have traditionally been classified based on their appearances as stubby (sessile), thin, and mushroom (pedunculated) types [12, 131]. In motoneuronal somatodendritic domain, the mushroom-shaped pedunculated spines were less frequent than the stubby (sessile) and thin and filopodia-like spines (Figure 1(c)), the sessile (stubby) spines being most common at P30.

In consistency with previous studies on other parts of the brain, such as the pyramidal cells from the rat visual cortex where transiently appearing filopodia mostly disappear after P12 [111, 132], there was a gradual developmental decrease in the filopodia density on the soma and dendrites of motoneurons at P15 (Figures 5(d) and 6(c)) and P30 (Figures 5(c) and 6(e)) compared to E17/P0 WT mice (Figures 3(c)-3(d), 5(a), and 6(a)-6(b)). This may coincide with the developmental downregulation of the GluR1 subunit of AMPA receptors, which has previously been shown to promote filopodia numbers in spinal motoneurons during postnatal development [133]. Spine density on the other hand increased, especially on the distal dendrites at P15 (Figures 5(d) and 6(c)), but then showed a decline at P30 WT mice (Figures 5(c) and 6(e)), especially on soma and proximal dendrites (Figure 6(e)). With maturation, the proportion of shorter and stubby spines on the soma and dendrites increased. One possibility is that some of the taller spine-like processes during early development may actually be shorter filopodia. However, longer filopodia-like processes were still present on distal dendrites, but not as common at P30 (Figure 6(e)). Other studies have also shown that higher levels of spine formation and loss occur in cortical and pyramidal cells from adolescent mice versus adults [13, 22, 111, 134]. During postnatal development, reduction in filopodia numbers in motoneurons may be subsequently followed by a reduction in spine numbers. The reduction in filopodia may limit the formation of new dendritic segments and synapses, promoting stabilized synaptic connectivity during transition to adulthood.

In mutant mice strains with impaired inhibitory synaptic transmission (lacking VGAT, GAD-67, or gephyrin), we saw an increase in the density and length of filopodia and spine-like structures on the soma and primary dendrites (Figure 5(b)) compared to WT littermates at E18-P0 (Figure 5(a)). Increases in density and length of filopodia and spine density may be a compensatory reaction to form functional GABAergic and glycinergic synapses. This also implies that the filopodia may also be involved in formation of GABAergic and glycinergic inhibitory synapses, not just excitatory glutamatergic synapses. There is evidence for GABA and glutamate corelease in the brain, and GABAergic synapses can be formed not only on dendritic shafts but also on dendritic spines of pyramidal neurons, where GABA may play a key role in the localized regulation of neuronal Ca^2+^ signaling [124]. It has been proposed that the active excitatory inputs may specifically attract (or repel) an inhibitory bouton; for example, GABAergic inputs may be recruited by the presence of specific glutamatergic afferents, as spines receiving a GABAergic synapse seem to be targeted by excitatory terminals expressing synaptic marker VGLUT2 [124]. We have shown that VGLUT2 is common in presynaptic terminals to motoneurons (Figure 3(d)), raising the possibility that similar mechanisms may also be implicated during motoneuron development and synaptogenesis. By increasing the length and density of filopodial and spine-like processes, and dendritic branching, motoneurons may be trying to increase their chances of finding GABAergic and glycinergic terminals. Given that the majority of synaptic contacts are found on filopodia of spinal motoneurons during early development [43, 135] (Figure 3(d)), it is possible that, after a long period of nascent inhibitory synaptogenesis, these filopodial processes are ending up with extra excitatory synapse formations. This is consistent with patch-clamp recordings showing significant decrease in the frequency and amplitude of inhibitory postsynaptic currents and potentials and a significant increase in the frequency of excitatory postsynaptic currents and potentials in mutant mice compared to their WT littermates (Figure 4). These findings fit with significant increases in hypoglossal nerve output in brainstem-spinal cord preparations of gephyrin knockout mice, compared to WT littermates [127]. Unfortunately, mice deficient in inhibitory synaptic transmission (GAD-67-KO, VGAT-KO, and gephyrin-KO) die soon after birth [127], preventing the acquisition of data past P0 from any of these mutants. Interestingly, mice overexpressing the mutated hSOD1^G93A^ gene, widely used as a mouse model of inherited ALS, also show increased density of filopodia and spine-like structures in their soma (Figures 5(e)-5(f)) and dendrites (Figures 6(d) and 6(f)) at P15–P30 of presymptomatic age, compared to WT littermates at P15 (Figures 5(c) and 5(d)) and P30 (Figures 6(c) and 6(d)). As for mice deficient in inhibitory synaptic transmission, these morphological changes correlate with increases in the frequency of EPSCs in motoneurons from mice overexpressing the hSOD1^G93A^ gene (Figure 7).

We have found that increased filopodia and spine-like processes were usually associated with increased dendritic length and branching in hSOD1^G93A^ mutants and inhibitory synaptic transmission deficient strains of mice including gephyrin, GAD67, and VGAT knockout mice compared to WT littermates. In WT mice, dendritic growth and branching and increases in spine density continued until about P15, when the filopodia density was high; after loss of filopodia, the growth of the motoneuronal dendritic length and branching, as well as increases in dendritic spine density, were reduced. Taken together these findings suggest that at least some of the filopodia may be involved in dendritic lengthening and branching and increased spine density during normal motoneuron development and that exaggerated filopodial formation leads to increased structural responses in hSOD1^G93A^, gephyrin, GAD67, and VGAT mutants compared to WT mice. The remaining fewer filopodia in WT adult motoneurons may reflect a residual regeneration capacity of motoneurons, while increases in filopodial number following injury or under neuropathological conditions may reflect reactivated capacity of motoneurons to regenerate.

The mechanisms driving increases in filopodial and spine density and shape (i.e., shortening and thickening), as well as dendritic branching and length, during normal development and under neuropathological conditions or injury are not clear. It is likely that multiple factors are involved: including innate genetic factors, neurotrophic and growth factors, hormones (e.g., androgens and estrogens), guidance cues, neuronal cell adhesion molecules (NCAMs), extracellular matrix, and neuronal activity regulated by the excitatory and inhibitory synaptic inputs and intrinsic membrane properties. Each of these factors will be discussed in detail below.

Brain-derived neurotrophic factor (BDNF) is a strong neuroplasticity candidate that can transform functional activity into morphological changes and dendritic complexity and stability, either during development or as a consequence of changed neuronal activity [20, 136–138]. BDNF can be released from neuronal dendrites or axons in response to neuronal activity or activation of glutamate receptors [116, 136, 138, 139]. BDNF acts upon tyrosine kinase B (or tropomyosin-related kinase B; TrkB) receptors or other signaling pathways such as serum inducible kinase (SKN) to regulate dendritic complexity, filopodial and spine density, and stability of these structures [20, 116–118, 137, 138, 140]. TrkB receptor signaling pathways are well characterized and involve the activation of rat sarcoma/extracellular signal-regulated kinases (Ras/ERK), phosphatidylinositol 3-kinase (PI3K)/Akt (protein kinase B), mitogen-activated protein kinase (MAPK), and phospholipase C- (PLC-) gamma pathways [117, 137]. MAPK and PI3K play crucial roles in translation and/or trafficking of proteins induced by synaptic activity, whereas PLC-gamma regulates intracellular Ca^2+^ that can drive transcription via cyclic adenosine monophosphate (cAMP) and protein kinase C (PKC) [117, 137]. In the neuromuscular system, androgens regulate BDNF and TrkB expression levels in spinal motoneurons and BDNF levels in target muscles [140, 141]. Androgen-BDNF interactions have important implications in motoneuron dendritic morphology [140, 141], possibly involving cytoskeletal proteins such as actin and tubulin. Castration results in decreased BDNF-TrkB and subsequent regression in dendritic morphology [140, 141]. Estrogens can also have significant effect on motoneuron dendritic growth during early postnatal development [142]. Estrogens are also known to interact with BDNF and to play important roles in brain neuroplasticity [143]; however details of this interaction have not yet been explored in regard to motoneuron plasticity [142]. Activation of BDNF-TrkB complex has been implicated in motoneuron vulnerability to SOD1^G37R^ mutations and toxicity [144]. After spinal cord injury, treadmill training induced lumbar motoneuron dendritic plasticity and functional recovery have been related to an increase in BDNF expression [145]. It has been reported that glutamatergic and GABAergic synapses react differently to postsynaptic BDNF: glutamatergic synaptic inputs increase, whereas GABAergic inputs decrease in a TrkB receptor-dependent manner [146].

NMDA receptors were originally considered to be the sole source of glutamate-mediated Ca^2+^ influx. However, AMPA receptors lacking developmentally regulated GluR2 subunit also allow a significant influx of Ca^2+^ ions [124, 147]. AMPA receptors also contribute to Ca^2+^ signaling by depolarizing the membrane, which activates voltage-gated calcium channels and relieves Mg^2+^ block from NMDA receptors [124]. Calcium influx activated by glutamate acting via AMPA/kainate receptors has been shown to have distinct and specific effects on the growth and development of motoneuron dendrites in E15 rat embryos [147]. This study suggested a potential physiological role for excitatory neurotransmission in dendrite growth and morphology during development when synaptic contacts are forming between afferent neurons and spinal motoneurons [147]. Increased glutamatergic synaptogenesis in other neuronal networks is also thought to occur through alterations in Ca^2+^ dynamics and/or glutamate-dependent synaptic plasticity [8, 12, 148]. For example, Ca^2+^ influx mediated by AMPA and NMDA glutamate receptors promotes or restricts spine growth in a concentration-dependent manner [9, 149], while overactivation of neurons can elicit increases in spine number and structure [7]. The complexity of dendritic arbor and branching of spinal motoneurons are refined in an activity-dependent manner that is sensitive to blockade of NMDA receptors during postnatal development, but not during late-adult postnatal life [75, 150]. However, NR3B subunit NMDA receptor expression is upregulated in adult motoneurons, and overexpression of NR3B increases dendritic complexity and branching and filopodia numbers [151]. During early postnatal life, synaptic activity promotes dendrite elaboration and growth in spinal motoneurons utilizing GluR1-containing AMPA receptors [152]. Overexpression of the AMPA receptor GluR1 subunit also resulted in an increase in filopodia numbers and in dendritic length and arbor complexity [133]. In contrast, AMPA receptor GluR2 overexpression did not alter dendritic complexity but was associated with increased arbor length and decreased filopodia numbers [133]. These authors concluded that downregulation of GluR1 during postnatal development might limit the formation of new dendrite segments and synapses, promoting stabilized synaptic connectivity with maturation [133]. Other studies have suggested a role for activity-regulated cytoskeletal associated protein involvement in NR2A subtype NMDA, GluR1 and GluR2 type AMPA receptor-mediated chronic spontaneous functional recovery of the paralyzed diaphragm muscle following cervical spinal cord hemisection [153].

Using genetic manipulations of plasma membrane K^+^ channel expression in* Drosophila*, it has previously been shown that increased intrinsic neuronal excitability can cause increased dendritic branch formation, whereas decreased intrinsic excitability can cause increased dendrite branch elongation of motoneurons [154]. Therefore, changes in dendritic complexity and plasticity can also be driven by alteration of intrinsic membrane excitability, independent of excitatory glutamatergic synaptic inputs. Since increases in excitatory synaptic inputs were common to motoneurons in all mutant mice used in our studies (Figures 4 and 7), we think it is likely that increases in dendritic branching, filopodia, and spine numbers reported here in mice overexpressing hSOD1^G93A^ and in mice with impaired inhibitory synaptic transmission (VGAT, GAD-67, and gephyrin knockouts) may be driven by increases in glutamatergic synaptic inputs onto motoneurons. Therefore, glutamate- and activity-dependent pathways are likely to be major players in structural remodeling of dendrites, spines, and filopodia under these neuropathological conditions.

However, the signal transduction mechanisms linking glutamate receptor activation to intracellular effectors that accomplish structural and functional plasticity are not well understood. Ultrastructural studies have revealed that the postsynaptic density (PSD) is a highly organized structure that scaffolds the receptors (such as NMDA and AMPA), enzymes, and signaling molecules required for synaptic transduction [16, 20, 155]. Spines are enriched with a complex network of actin, termed the actin spinoskeleton, which supports and determines the physical structure and shape of the dendritic spine [20, 155]. The actin spinoskeleton also organizes the postsynaptic signaling machinery, drives changes in spine structure, and maintains spine stability [16, 20, 156]. The highly dynamic actin cytoskeleton is regulated by an abundance of actin-binding proteins and upstream signaling pathways that modulate actin polymerization and depolymerization [109]. A long list of actin-binding proteins includes actin depolymerizing factor (ADF)/cofilin, actin-related proteins 2/3 (Arp2/3) complex, brevin, calpactin, cortactin, calcium/calmodulin-dependent protein kinase II (CaMKII), calponin, cofilin, drebrin, dystrophin, epidermal growth factor (EGF) receptor, ERM proteins, gelsolin, G-proteins, myosins, myelin basic protein, neurexins, plastin, phalloidin, PKC, rapsyn, suppressor of* ras* ^*Val14*^ (srv2), synaptopodin, spectrin, tau, tropomyosin, and Wiskott-Aldrich syndrome protein (WASP) [109]. Glutamate receptor activation is one of the most characterized regulators of actin-based dendritic spine plasticity [16, 20, 157, 158]. CaMKII-dependent phosphorylation of kalirin-7 and subsequent activation of the small GTPase Rac1 are required for NMDA receptor-dependent rapid changes in spine morphology (i.e., enlargement of existing spines) and GluR1 AMPA receptor insertion into synapses of pyramidal neurons [159]. Inactive CaMKII can bind F-actin, thereby limiting access of actin-regulating proteins to F-actin and stabilizing spine structure [156]. Activation of CaMKII dissociates CaMKII from F-actin and permits F-actin remodeling by regulatory proteins followed by reassociation and restabilization [156]. Thus CaMKII kinase can regulate a transient interplay between the kinase and structural functions during the induction of synaptic plasticity.

Recent studies have also identified a neuronal cell adhesion molecule, Dscam1 (Down's syndrome cell adhesion molecule 1), to be critical in regulating developmental dendritic arbor growth, spine and synapse formation, and circuit wiring in mice cerebral cortical and hippocampal pyramidal neurons [160, 161] and in dendritic arbor growth and branching of* Drosophila* motoneurons [162, 163]. Significant loss of Dscam1 function in* Drosophila* prevented stable dendrites from being formed and mutant motoneurons were devoid of mature dendritic branches, instead of displaying a dense meshwork of filopodia-like and lamellipodia-like processes, normally seen during early pupal development [162]. Therefore, Dscam1 may be required for the transformation of actin-rich highly dynamic filopodia into stable dendrites, although the underlying mechanism remains to be elucidated [162]. It is possible that Dscam1 affects intracellular signaling pathways, such as small Rho GTPase, which regulate dendritic growth cone dynamics and spine formation and stability. The intracellular domains of* Drosophila* and human Dscam interact with P21-activated kinase (PAK1), which, in turn, provides a possible mechanistic link to Rho GTPases and actin polymerization [162]. Dendritic translation of Dscam1 is regulated by NMDA receptor activation, and impairment of NMDA-mediated regulation of Dscam1 has been implicated in alterations in dendritic morphology and synaptic plasticity in Down's syndrome [160]. Surprisingly, Dscam1 deficient* Drosophila* flight motoneurons that lack 90% of their dendrites but have normal axonal structure and membrane currents can still satisfactorily perform the vast majority of basic motor functions [163]. Motoneurons with significant dendritic defects can still be contacted by appropriate synaptic partners and can produce qualitatively normal firing patterns and wing movements during flight and courtship song behaviors [163]. However, a normal complex 3D dendritic architecture is essential for intricate regulation and fine tuning of behavior and particularly challenging tasks, such as the integration of optomotor input for adequate control of flight power output or the temporal accuracy of switching between different song elements during courtship to ensure mating success [163].

In vertebrates, cadherins and catenins are the major cell adhesion molecules involved in regulation of dendritic branching and synaptic morphogenesis [164, 165]. The cadherins are glycosylated transmembrane proteins associated with a group of cytosolic proteins, the catenins, and they form cell adhesion complexes in various tissues [165]. A recent study showed that the spine pruning and maturation in the mouse somatosensory cortex are coordinated via the cadherin/catenin cell adhesion complex and bidirectionally regulated by sensory experience [166]. This study concluded that activity-induced interspine competition for beta-catenin provides specificity for concurrent spine maturation and elimination and thus is critical for the molecular control of spine pruning during neural circuit refinement as well as under neuropathological conditions such as autism [166]. The cadherin family is composed of more than 100 members and classified into several subfamilies, including classical cadherins and protocadherins. Protocadherins constitute the largest cadherin family, with 68 members in humans and 70 in murine [165]. Inhibition of cadherin function in cultured hippocampal neurons using a dominant negative approach resulted in abnormal morphogenesis of spines, including filopodia-like elongation and spine head bifurcation, along with disruptions at postsynaptic and presynaptic proteins and synaptic vesicle recycling [164]. In the spinal cord, early studies showed that protocadherin gamma proteins were required for survival of spinal interneurons, synaptic development, and maturation of spinal neurons [167]. Mice lacking all 22 genes of the protocadherin gamma cluster have decreased numbers of spinal cord synapses, are nearly immobile, and die shortly after birth [167]. More recent study showed that protocadherins were involved in mediating dendritic self-avoidance process, in which branches arising from a single neuron repel each other, in the mammalian retinal starburst amacrine interneurons and cerebellar Purkinje cells, mirroring those reported for Dscam1 function in* Drosophila* mentioned above [168]. The clustered protocadherins regulate neuronal survival, as well as dendritic self-avoidance. Nonclustered protocadherins promote cell motility rather than the stabilization of cell adhesion, unlike the classic cadherins, and mediate dynamic cellular processes, such as growth cone migration [165, 169]. Cadherin superfamily members are implicated in several neuronal disorders including Alzheimer's disease, schizophrenia, autism, mental retardation, and epilepsy [165, 169].

Cell adhesion molecules also connect to both the presynaptic partner and the extracellular matrix (ECM), which is composed of glycoproteins (e.g., laminins, tenascins, and thrombospondins) and proteoglycans that form a complex interactive meshwork in and around the synaptic cleft [170]. The neurons, glial cells, and the space adjacent to and between synapses are surrounded by ECM providing a “glue or gel” to attach cells and processes to each other [170]. At the synaptic cleft, pre- and postsynaptic cell adhesion molecules associate with one another and with the ECM to initiate and maintain synaptic contact [170]. These transmembrane cell adhesion proteins connect to the intracellular dendritic spine actin network and influence the activities of actin regulatory molecules, thereby controlling spine shape. The ECM has therefore been implicated in spine and synapse stability, remodeling, and plasticity during development and adulthood [170]. These are important functions as loss of spine stability has been implicated in a number of neurodegenerative diseases [170].

ECM also attaches signals with special domains docking to cell surface receptors and presents soluble molecules such as basic fibroblast growth factors (bFGFs) or wingless/Int-1 (Wnt)—proteins critical for neuronal survival and identity determination [92]. The availability of these molecules depends on the matrix composition and influences the transcription factor code of each cell. Recent research has also provided strong evidence that depletion of single matrix molecules like Tenascin C (TnC) can lead to developmental changes within the motoneuron progenitor pools [92]. Modulation of pathways involving potently inhibitory ECM may be critical in recovery from spinal cord injuries [171]. Following spinal injury, digestion with chondroitinase ABC of the upregulated chondroitin sulphate proteoglycans, that restrict functional plasticity and stabilize spines, is beneficial in motoneuronal plasticity and functional recovery of paralyzed diaphragm [171].

Upon neuronal activation or stimulation, the actin spinoskeleton is uniquely regulated within microdomains to modulate spine morphology, PSD structure, and membrane trafficking that involves the dynamic processes of exocytosis, endocytosis, internalization, endosomal recycling, and localization of molecules such as AMPA receptors important in synaptic transmission and neuroplasticity [172]. Actin dynamics generate forces that manipulate membranes in the process of vesicle biogenesis and also for propelling vesicles through the cytoplasm to reach their destination [172]. In addition, trafficking mechanisms exploit more stable aspects of the actin cytoskeleton by using actin-based motor proteins such as myosins to traffic vesicular cargo along actin filaments [172, 173]. Myosins are a large family of actin-based cytoskeletal motors that use energy derived from adenosine triphosphate (ATP) hydrolysis to generate movement and force for functions such as regulation of actin cytoskeleton dynamics in dendritic spines and powering of synaptic cargo transport [173]. In summary, mounting evidence indicates that the actin molecule with rapid dynamics is at the centre stage of structural regulation, maintenance, and remodeling of synaptic plasticity [16, 20, 155, 156].

Recent studies have found that alterations or disturbances in the cytoskeletal actin pathway in motoneurons and redox alterations in the inflammatory compartment contribute to ALS pathogenesis and disease outcome [174–178]. Profilin 1 (PFN-1) is crucial for the conversation of monomeric (globular) G-actin to polymer microfilament (filamentous) F-actin in response to extracellular signals [109], and mutations in PFN-1 gene are shown to cause familial ALS [175, 177]. Primary motoneurons expressing mutant PFN-1 display smaller growth cones with a reduced F/G-actin ratio [175]. Actin-binding protein plastin 3 (PLS-3) levels are reduced in spinal muscular atrophy (SMA), and transgenic reintroduction of PLS-3 rescues functional defects in SMA [174, 178]. SMA is due to gene mutations or deletions in the survival motor neuron 1 (*smn1*) gene, decreasing the availability of SMN protein, which in turn leads to an early degeneration of lower motor neurons in children [178]. SMN protein regulates actin dynamics, and SMN overexpression in cultured neuronal cells promotes neurite outgrowth [174, 176, 178]. Small GTPase Rac1 dysregulation or alterations in structure and function have also been implicated in the pathogenesis of ALS and SMA [176]. Rac1 plays a key regulatory function of both actin and microtubule cytoskeletal dynamics and thus it is central to axonal and dendritic growth and stability, as well as dendritic and spine structural plasticity during development and under neuropathological conditions. Rac1 is also a crucial regulator of nicotinamide adenine dinucleotide phosphate-oxidase- (NADPH-) dependent membrane oxidase (NOX), a prominent source of reactive oxygen species (ROS), thus having a central role in the inflammatory response and neurotoxicity mediated by microglia [176]. SOD1 directly binds to Rac1 in a redox-sensitive manner: in reducing conditions SOD1 binds to Rac1 and stimulates its activity; conversely, in oxidizing conditions SOD1 dissociates from Rac1 and inhibits its activity [176].

Figure 7 illustrates how these developmental and neuropathological changes (in the case of hSOD1^G93A^-mutated mice) in dendritic, filopodial, and spine morphology are reflected in functional properties of motoneurons, as recorded in the form of excitatory postsynaptic currents (EPSCs) received by the motoneurons. At all age groups, in particular at P15 and P30, the frequency of EPSCs is significantly higher in large-sized hypoglossal motoneurons from hSOD1^G93A^ transgenic mice (Figure 7(b)), compared to age-matched WT mice (Figure 7(a)). Amplitude of EPSCs was slightly larger in hSOD1^G93A^ overexpressing motoneurons than WT motoneurons, but not as dramatic as changes in EPSC frequency (Figure 7). This suggests that changes at the synaptic level are primarily due to increases in the total number of glutamatergic excitatory synapses on to hSOD1^G93A^ overexpressing hypoglossal motoneurons. This is consistent with denser filopodia and spine numbers in motoneurons from hSOD1^G93A^ mice (Figures 5(e), 5(f), 6(d), and 6(f)) compared to WT (Figures 5(a), 5(c)-5(d), 6(a)–6(c), and 6(e)).

In WT motoneurons, there is an increase in motoneuron EPSC amplitudes associated with a decrease in frequency during motoneuron development (Figure 7(a)), coinciding with decreases in dendritic spine numbers and transition to shorter and stubby spines (Figures 5(c)-5(d), and 6(e)). Indeed, these size and shape changes (i.e., shortening and increase in thickness) in spines and filopodia during postnatal development may reflect the strengthening of synapses in an activity-dependent manner, as shown for the pyramidal neurons [20, 111, 179]. In particular, stubby spines seem to be much more stable and persistent than longer and thinner spines [179], and filopodial processes that are much more dynamic than dendritic spines may also be less stable [111, 179]. These developmental changes in the size and density of spines and increases in the amplitude of EPSCs reported here suggest an increase in synaptic strength and efficacy with maturation of motoneurons. Spine size correlates with synaptic strength and larger spines commonly contain larger PSDs with more AMPA-type glutamate receptors and appose axon terminals with larger readily releasable pools of neurotransmitter [6, 170, 179]. Therefore, the presence of stubby and large diameter spines is more likely to produce strong excitatory postsynaptic currents and has greater influence on neuronal firing and network signaling. While small amplitude excitatory synaptic inputs will need to summate to cause firing, a large amplitude excitatory synaptic input from a single presynaptic fiber can trigger action potentials in the postsynaptic neuron [180, 181]. As excitatory synaptic strength increases, the motoneuron may receive fewer but larger amplitude (stronger) excitatory synaptic inputs to bring the membrane potential at the axon initial segment to firing threshold by activating voltage-gated sodium channels. Riluzole-sensitive persistent sodium current present in motoneurons would be expected to further assist these large synaptic depolarizations [26, 182]. This makes sense as motoneurons, once activated, often have an intrinsic tendency to fire tonically in rhythmic bursts of relatively low frequencies (0.5–2 Hz burst frequency), interrupted by inhibitory inputs and after hyperpolarizations, such as during walking and breathing [33, 68, 69, 98, 183–185].

In pyramidal and Purkinje cells, spines do not only appear and disappear, but their basic morphology also seems to change continuously [9, 157, 186], often in an activity- or experience-dependent manner [10, 14, 17, 22, 187–191]. Although glutamatergic pyramidal cells and GABAergic Purkinje cells are very different in their morphology and function from cholinergic motoneurons, similar molecular mechanisms of plasticity, such as dynamic actin-based cytoskeletal remodeling of dendritic spine structure, size, and shape, can also take place in motoneurons in response to experience and activity or under neuropathological conditions. Such dynamic plasticity of sensory- and activity-dependent development of the neuromotor system and the modifications that take place after disease or injury have been the focus of many studies [18, 76, 127, 145, 178, 192–194].

## 4. Spinogenesis/Synaptogenesis and Plasticity during Motoneuron Development

As most spines are believed to be postsynaptic compartments receiving excitatory synaptic inputs [8], spinogenesis is linked to synaptogenesis [12]. An initial period of spine proliferation during early development is probably intrinsic to the neuron, as spines can emerge in the absence of axon terminals; the activity of the synapse and the neuron regulates a later decline in spinogenesis [12, 190]. A large proportion of spinogenesis and synaptogenesis in the primary visual cortex occurs before eye opening in mice, and the only morphological event that seems to correlate with eye opening is the elongation of the spine neck [132]. We also regularly observe filopodia and spines on growth cones and dendritic processes of developing human neurons derived from induced pluripotent stem (iPS) cells, in culture prior to formation of synaptic inputs (Figures 1(a)-1(b)). Therefore it is likely that the sensory input or sensory evoked activity and axonal input are not essential for ontogenetic spinogenesis/synaptogenesis in neurons, such as visual cortical neurons, during embryonic/prenatal or even neonatal period [12, 13, 132, 190]. Instead the robust spontaneous activity of the developing brain and spinal network* in utero* is potentially important for normal spinogenesis [12, 13, 132, 190]. However, the spine stability and modifications to spine shape and size can be strongly modulated by sensory manipulations and activity [13, 20, 22, 24, 189–191, 195, 196]. Previous studies have shown spine [7] and filopodia [197] generation after induction of synaptic potentiation using two-photon laser microscopy. Using similar methods in mice* in vivo*, others have shown that spines are remarkably stable throughout life in cerebral cortical neurons [14, 15, 111, 134, 179, 189, 191, 198], including primary visual cortical layer 5 pyramidal neurons in the primary visual cortex of living transgenic mice expressing yellow fluorescent protein [111, 134]. The turnover of spines is higher during the critical period of postnatal development than in adult life [13, 22, 111, 134]. These* in vivo* studies also found that spines undergo considerable changes in shape and size during development and in the adult mice, as previously shown in* in vitro* preparations [10, 134, 157, 158].

Numerous cell surface receptors, scaffold proteins, and actin binding proteins present in spines engaged in spine morphogenesis [156, 199–204]. Molecular studies indicate that multiple signaling pathways, particularly those involving Rho and Ras families of small GTPases, such as Rac1, RhoA, and cdc42, play important roles in morphogenesis of dendritic spines, spinogenesis, spine loss or retraction, and synaptic plasticity, by converging on the actin cytoskeleton to bidirectionally regulate spine morphology and dynamics [199–204]. Overexpression of these small GTPase proteins results in the creation of new spines* in vivo* and* in vitro* in Purkinje cells and pyramidal neurons [199, 200, 205]. Spine density, size, and length are controlled by different members of the Rho and Ras families in developing and mature Purkinje cells and pyramidal neurons [201–205]. Therefore even mature neurons have the entire molecular complement required for spinogenesis in response to injury and neuropathological conditions.

Spine stability is most likely due to binding of inactive CaMKII to F-actin, thereby limiting access of actin-regulating proteins to F-actin [156], as well as contribution from cell adhesion molecules and the extracellular matrix [170]. Activation of CaMKII by sensory stimulation or synaptic activity may be important in altering the structural stability of actin and spines [156]. This role is critical as most brain disorders and neurodegenerative diseases including Alzheimer's, Parkinson's, and Huntington's diseases and schizophrenia involve loss of dendritic spine stability in adulthood [170].

**Figure 8:**
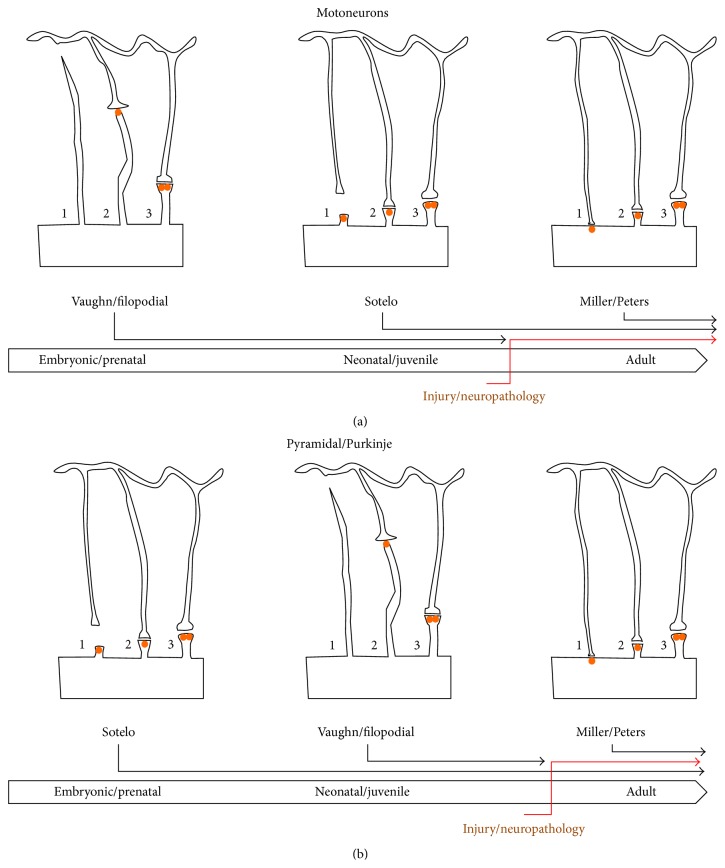
A schematic presentation of a unified-hybrid model of spinogenesis/synaptogenesis in motoneurons compared to pyramidal/Purkinje neurons at different developmental stages involving previously described models. (a) In motoneurons, the Vaughn/filopodial model (left) predominates during embryonic, prenatal, and neonatal development but becomes less common during juvenile development, where the Sotelo model (middle) becomes more frequent. The Miller/Peters model (right) may play a role in adult plasticity. (b) The sequence of spinogenesis/synaptogenesis in motoneurons seems to significantly differ from that described for pyramidal and Purkinje cells. The Sotelo model predominates in spinogenesis in pyramidal/Purkinje cells during embryonic/prenatal period. The filopodial model is less involved in all cell types in the adult but is likely reactivated as part of the regenerative/remodelling processes following injury/neuropathological conditions (red lines). 1, 2, and 3 indicate the sequence of pre- and postsynaptic development; filled circles represent postsynaptic receptor clusters.

Despite our increasing understanding of the molecular players in spinogenesis, the mechanism of spine formation is still widely debated. Three major models of spinogenesis in the nervous system have been put forward by previous studies [3, 12]: these are proposed by Miller/Peters [132], by Sotelo [206, 207], and by the filopodia model derived from Vaughn's synaptotropic hypothesis [43–46] (Figure 8). The Miller/Peters model is based on Golgi-electron microscopic data from developing rat visual cortex pyramidal cells; in this model, protrusions with a stubby appearance are first formed on dendritic shafts; then an apposed presynaptic region of the axon shows a swelling as synaptic vesicles accumulate, inducing the dendritic shaft to initiate elongated spine formation; in the final stage (at around P21 in mice), many spines are thin or mushroom shaped [132]. However, more recent studies have found that dendritic spines can grow directly from a dendritic shaft in contact with an axon which induces the clustering of Rac1, a small GTPase from Rho family involved in dynamic regulation of actin and the microtubule cytoskeleton, leading to spinogenesis [208], so that spine formation precedes synapse formation [190, 209]. The Sotelo model, based on data from cerebellar Purkinje cells of normal and mutant mice, contradicts the Miller/Peters model; as Purkinje cells from mutant mice lacking parallel fibers form abundant spine, the Sotelo model proposes that the presynaptic axonal terminal has only a minor role in spinogenesis [206, 207].

The filopodia model is derived from Vaughn's synaptotropic hypothesis, based on ultrastructural and Golgi studies on embryonic and neonatal mice spinal cord motoneurons [43–46]. The filopodial model posits that spines arise by transformation of existing filopodial precursors or protospines, as has been shown by live confocal time-lapse imaging studies [47, 129]. Studies of hippocampal and cortical pyramidal neurons have provided supporting evidence for this model by confirming that existing filopodia can produce both sessile and pedunculated spines [47, 129, 130].

A unifying model for synaptogenesis during different developmental stages has recently been put forward by García-López et al. [3], which incorporates elements of these previous models and proposes that synaptogenesis may happen in three different modes, corresponding to prenatal (Sotelo model), neonatal (filopodial model), and mature (Miller/Peters model) age groups, respectively (Figure 8). Our data spans this developmental spectrum, and our observations fit most closely with the filopodial model during embryonic/prenatal/neonatal periods; although Sotelo model may also contribute particularly in neonatal/juvenile period, Miller/Peters model is likely involved primarily in adults. The unified model also overlaps with our results obtained from developing motoneurons. However our motoneuron-based model also differs from the unified model [3] in the sequence of events, with filopodial model, instead of Sotelo model, being dominant during embryonic/prenatal/neonatal periods; then it is followed by involvement of Sotelo model at neonatal/juvenile periods, and the Miller/Peters model may get involved much later at juvenile/adult stages (Figure 8). Our data from embryonic/neonatal/juvenile motoneurons is least consistent with the Miller/Peters model based on pyramidal cells, as this model may be more applicable to spinogenesis/synaptogenesis in adult motoneurons (Figure 8). Spinogenesis in motoneurons therefore seems to have a uniquely sequenced unified-hybrid model differing in sequence of events from previous models based on pyramidal and Purkinje neurons, as summarized in Figure 8. This may be due to major developmental differences between the pyramidal/Purkinje cells and hypoglossal/lumbar motoneurons. These include the following. (1) Motoneurons are cholinergic, receive excitatory glutamatergic synaptic inputs before birth, and are inhibited by GABA and glycine prior to birth (Figure 4); by contrast, pyramidal neurons do not have excitatory glutamatergic synapses until after birth and are excited by GABA during prenatal and neonatal periods. (2) Filopodia and long thin spine-like processes appear on embryonic motoneurons forming synapses (Figures 3(c)-3(d), 5(a), and 6(a)-6(b)), as previously shown [43, 44]. We have seen filopodia on motoneuron soma and dendrites at the earliest age that we have examined (E16), and filopodia likely appear earlier than that, as others have shown synapses forming on dendritic filopodia as early as E11 in mice spinal motoneurons [44, 46]. Indeed, around 70% of synaptic contacts are found on filopodia in the developing mice [43] and chicken [135] spinal cord motoneurons. By contrast, in pyramidal cells, filopodia of the collateral type are transient and appear later postnatally at P3–P12 and rarely form synapses [132]. Synapses on dendritic shafts predominate in pyramidal cells in early development, with spine formation beginning in the first week after birth [132, 210]. (3) In motoneurons, filopodia and spine-like processes are thin and longer at late embryonic and early postnatal ages, becoming shorter and stubby with postnatal development, and mushroom spines are rare (Figures 5(c)-5(d), 6(c)–6(e)). In cerebral pyramidal neurons during early postnatal development, short and stubby spines are common, while in the adult, thin and mushroom spines are more common, although many stubby spines are still present [132, 211]. (4) Pyramidal and Purkinje neurons are innervated by regular parallel fibers, by contrast to developing presynaptic axon terminations within the hypoglossal nucleus following convoluted routes with many three-dimensional twists and turns in mouse and rat (Kanjhan, unpublished observations), as shown for the adult rat [69]. This suggests that formation of synapses on filopodia during development follows contact with searching axon terminations (Figure 3(d)) [43, 44, 135], before being transformed into dendritic spines or incorporated into the dendritic shaft by filopodial retraction. Filopodia contacting several axons can distinguish between distinct inputs and choose the most active ones [3].

It is possible that cholinergic motoneurons, which extend long axonal projections to muscles in the periphery and which receive descending premotor and local interneuron inputs, use different spinogenesis strategies than inhibitory (GABAergic) Purkinje cells and excitatory (glutamatergic) pyramidal neurons. One feature that supports this is that spine shapes in different cell types (e.g., pyramidal, Purkinje, and motoneurons) fall into different dominant categories. In the somatodendritic domain of mature motoneurons (P30), stubby and short type spines are seen more frequently than the relatively sparse longer mushroom-shaped spines, which are the dominant spine shape in adult pyramidal and Purkinje cells [12, 131, 132, 211]. While motoneuron spines get shorter (stubby) with maturation, in contrast the pyramidal and Purkinje neuron spines seem to be getting longer. Unfortunately there are no specific markers, which can distinguish different spine types or differentiate spines from filopodia. The filopodial model of spinogenesis, which is relatively less active in juvenile/adult period, may become reactivated following neuronal injury/neuropathological events as part of the structural regenerative remodelling of the neuronal morphology (Figure 8).

We have noted differences in filopodia and spine density between motoneurons from the same slice preparation, as well as between the dendrites of the same motoneuron. Morphological differences, such as spine density and shape (short and stubby spines) and dendritic branching, among categories of motoneurons may also be important in providing structural bases for synaptic hyperexcitability leading to neuropathological conditions, such as selective degeneration of certain types of motoneurons (e.g., vulnerable F type phasic large alpha motoneurons) in ALS [89, 106, 107, 194, 212–214]. The potential neuropathological roles of morphological changes will be discussed below.

## 5. Excitatory Hypersynaptogenesis and Intrinsic Membrane Hyperexcitability Are Linked to Insufficiencies in Cellular Energy Metabolism and Selective Motoneuron Degeneration

Amyotrophic lateral sclerosis (ALS) and spinal muscular atrophy (SMA) are neurodegenerative disorders characterized by selective loss of motoneurons, most likely due to cellular excitotoxicity and oxidative stress caused by accumulation or deregulation of intracellular Ca^2+^ levels resulting primarily from glutamate receptor activation [107, 178, 193, 194, 212–218]. Elevated intracellular Ca^2+^ can activate cytoplasmic apoptotic proteins such as calcineurin and calpain, deregulate of Ca^2+^ in the endoplasmic reticulum, and overload the mitochondria with Ca^2+^, resulting in mitochondrial dysfunction and oxidative stress ultimately promoting neuronal death [216, 218, 219]. Most cases of ALS are sporadic with no known genetic linkage, while approximately 10% are associated with familial forms, presenting mutations in over 20 genes encoding for distinct proteins with varied functions, including SOD1, fused in sarcoma (FUS), TDP-43, chromosome 9 open reading frame 72 (C9ORF72), PFN-1, vacuolar protein sorting-associated protein 9- (VPS9-) ankyrin repeat protein (VARP), alsin, ataxin-2, and matrin-3 [194, 218]. Mutations in these proteins may increase the susceptibility for the dysregulated intracellular Ca^2+^-mediated degenerative processes to occur, suggesting existence of a common pathogenic pathway centered around intracellular Ca^2+^ and its handling [194, 216, 218]. For example, misfolded and aggregated SOD1 mutants localized within the mitochondrial membrane of spinal cord motoneurons cause dysfunction in oxidative phosphorylation and lead to endoplasmic reticulum stress [194, 216, 218, 219].

Consistent with glutamate-mediated excitotoxicity hypothesis, our studies of motoneurons from hSOD1^G93A^ mutant mice show that increases in dendritic spine density compared to age-matched littermates are associated with increased frequency of EPSCs, as well as an enhanced developmental increase in EPSC amplitudes (Figure 7(b)). Increased frequency of excitatory and inhibitory synaptic inputs to hypoglossal motoneurons, together with increased intrinsic persistent sodium currents resulting in increased action potential firing rates, was previously reported in hSOD1^G93A^ mutant mice as early as P4 to P10 [192]. These modifications in incoming excitatory synaptic inputs interact with changes in the intrinsic membrane properties of the motoneurons. For example, increases in persistent sodium and calcium currents may result in longer lasting depolarizations following excitatory synaptic inputs [97, 215, 217, 220], leading to excessive sodium and calcium loading of the cytoplasm and specific compartments such as spine heads. Such longer depolarizations may in turn reduce the excitability and firing properties of motoneurons, by depolarization block or partial inactivation of the voltage-gated sodium channels [217]. This may be the mechanism of hypoexcitability of lumbar motoneurons reported in adult hSOD1^G93A^ mutant mice [221]. Accumulation of intracellular calcium will also result in disturbances in calcium homeostasis and protein folding, endoplasmic reticulum stress [194, 212, 215, 217, 219, 222], and perturbations in the function and motility of the actin-based cytoskeleton and spinoskeleton [10, 16, 20, 155, 158, 174, 175, 177, 178, 203]. However, recent studies have questioned the role of hyperexcitability in motoneuron degeneration [36, 37, 221]. The excitability of large F-type motoneurons was unchanged in the mSOD1^G93A^ mutant neonatal mice, but, surprisingly, the small S-type motoneurons displayed intrinsic hyperexcitability [36]. Another study using two-photon imaging found that calcium transients in motoneuron dendrites of hSOD1^G93A^ mutants are smaller, compared to WT mice at P4–P11 [37]. These findings may not be conclusive, given the wide variability in their Ca^2+^ responses and the inability to measure the total Ca^2+^ entry into the cell. However, data presented here and overwhelming evidence from various labs around the world, including recent studies from human motoneurons derived from ALS patient induced pluripotent stem cells (iPS cells, see below), support a role for hyperexcitability in the development of motoneuron degeneration [91, 223].

A reduction or depletion of intracellular ATP will have further consequences on neuronal activity, by cyclical activation ATP-sensitive potassium (K^ATP^) channels that set burst frequency and duration in motoneurons [184, 215, 217]. Motoneuronal bursting under neuropathological conditions, such as inhibition of glutamate uptake by astrocytes, may involve persistent glutamatergic activation of NMDA, AMPA (GluR2 lacking Ca^2+^ permeable), and metabotropic glutamate receptor type-1 (mGluR1) receptors to cyclical activation of K^ATP^ conductances, linking electrical discharge properties to the cellular energy metabolism in motoneurons [215, 217]. Firing properties of motoneurons may be further boosted by activity-dependent increases in extracellular K^+^ levels (~6 mM) and decreases in Ca^2+^ levels (~0.9 mM) consequent to increased locomotion or hyperactivity [224].

Another intrinsic factor that is critical in motoneuron excitability is the relative contribution from various K^+^ channels including the delayed-rectifier K^+^ current (Kv7 or M-current) [225, 226] and TASK-1 two-pore domain leak K^+^ channels that are regulated by many extracellular and intracellular factors including several neurotransmitters such as glutamate, serotonin, and noradrenaline [33, 227, 228]. A reduction in K^+^ currents would increase the input resistance and the intrinsic excitability of motoneurons, potentially increasing the effects of excitatory synaptic inputs. Similar mechanisms have been implicated in a mouse model of neuronal atrophy in spinocerebellar ataxia type 1, involving abnormal membrane depolarizations due to a reduction in K^+^ channels, including TASK-1-like background K^+^ currents [229]. Postnatal increases in expression of TASK-1 channels likely dampen the excitability of motoneurons, perhaps serving to increase precision in muscle control and to reduce involuntary muscle contractions, as well as serving as a neuroprotector by letting K^+^ out of the cell in a voltage-independent manner [228]. Activation of glutamatergic metabotropic mGluR1 receptors inhibits TASK-1 like background K^+^ channels, subsequently increasing input resistance, motoneuronal excitability, and bursting activity [217]. Motoneurons may become more reliant on these TASK-1 leak K^+^ channels with aging [228], and changes such as increased inhibition of these channels via glutamatergic mGluR1 or peroxide-mediated oxidative stress may have undesired effects [217]. The role of K^+^ channels may become more critical when the extracellular K^+^ levels are raised to ~6 mM by increased locomotion in an activity-dependent manner [224]. Another important role is also played by astrocytes surrounding motoneurons, as they can clear extracellular K^+^ and glutamate in an activity-dependent manner using their ionic pumps (e.g., Na^+^-K^+^ ATPase, Na^+^-K^+^-2Cl^−^), K^+^ channels (e.g., inwardly rectifying K^+^ channels, especially Kir4.1), excitatory amino acid transporters EAAT1 (GLAST) and EAAT2 (GLT-1), and gap junctions made of connexins, Cx43 and Cx30 [230, 231].

Thus, reduced intracellular ATP availability increases the metabolic cost of a single action potential and disrupts K^+^ and Na^+^ homeostasis, resulting in a chronic depolarization and mitochondrial stress and dysfunction, which subsequently leads a cascade of events to selective degeneration of motoneurons [232]. Distinct subsets of motoneurons may also have variable bioenergetics needs. Motoneurons are large neurons with extensive dendritic trees and longest axonal projections requiring continuous and metabolically demanding transport of various molecules and mitochondria to the terminals [232]. Motoneurons are extremely active, continuously firing action potentials to maintain tonic posture or to generate the complex firing patterns needed for muscle contraction during specific movements, adding to the metabolic burden that must be met by ATP, produced both via oxidative phosphorylation and glycolysis [232]. Increased synaptic hyperactivity and longer lasting depolarizations will increase this metabolic demand, putting stress on mitochondria, as cellular Na^+^ and Ca^2+^ overloading increase energy use by many homeostatic exchangers, such as plasma membrane Na^+^-K^+^ ATPase (sodium pump), plasma membrane and sarcoplasmic Ca^2+^-ATPase (calcium pumps), and plasma membrane and mitochondrial Na^+^-Ca^2+^ exchangers to maintain the ionic homeostasis critical for neuronal survival [232]. Interestingly, the *α*1 isoform of Na^+^-K^+^ ATPase is differentially expressed in large alpha motoneurons, compared to small gamma motoneurons, which express the *α*3 isoform [233]. The *α*1 isoform of Na^+^-K^+^ ATPase extrudes intracellular Na^+^ at a slower rate than *α*3 [234], and this may well be a factor relevant to the selective degeneration of larger F-type alpha motoneurons in ALS patients and hSOD1^G93A^ mutant mice [89, 106, 107, 194, 214]. Na^+^-K^+^ ATPase is vulnerable to aberrant SOD1 activity, and global loss of Na^+^-K^+^ ATPase and its nitric oxide-mediated regulation occur in mice overexpressing hSOD1^G93A^ [235].

Defects in mitochondrial transport are implicated in the pathogenesis of several major neurological disorders [236]. Recent studies have identified mitochondrial Rho1 (Miro1) GTPases, a mitochondrial calcium sensor for glutamate receptor-dependent localization of mitochondria at synapses, as a key determinant of how energy supply is matched to energy usage in neurons [236, 237]. Trafficking of mitochondria to dendritic and axonal locations in neurons, where there are large Na^+^ and Ca^2+^ fluxes requiring active function of pumps, is essential for maintaining neuronal function and health. In fact, mitochondrial trafficking is regulated by Ca^2+^ flux through ionotropic glutamate receptors [237]. Miro1 links mitochondria to kinesin-1 family 5 (KIF5) motor proteins in a Ca^2+^-dependent manner (i.e., inhibited by micromolar Ca^2+^ binding to Miro1), allowing mitochondria to move along microtubules (anterograde or retrograde) until mitochondrial stopping induced by glutamate or neuronal activity [236, 237]. For example, activation of NMDA receptors leads to Miro1 positioning mitochondria at the postsynaptic side of synapses [237]. Miro1 is essential for development of cranial motor nuclei and maintenance of upper motor neurons, and neuron-specific loss of Miro1 causes depletion of mitochondria from corticospinal tract axons and progressive neurological deficits [238]. Defects in Miro1-mediated mitochondrial motility and distribution are sufficient to cause neurological disease such as upper motoneuron disease [238]. A significant reduction in Miro1 levels in the spinal cord tissue of ALS patients and transgenic models of ALS (SOD1^G93A^, TDP-43^M337V^) was recently shown by immunoblot analysis [239]. The same study also showed that excessive glutamate challenge leads to a significant reduction in Miro1 expression in spinal motoneurons of mice, suggesting that glutamate excitotoxicity may cause Miro1 deficiency leading to motoneuron degeneration [239].

These studies together suggest that excessive glutamatergic synaptic activity and changes in intrinsic membrane properties leading to sustained membrane depolarization will increase the energy demand of a motoneuron. If the supply of energy falls behind the consumption of energy required to maintain physiological levels of cytoplasmic Ca^2+^ and Na^+^, the rise in the intracellular levels of these ions will activate process that will lead to motoneuron degeneration.

Recent developments in stem cell technologies have allowed generation of human motoneurons from somatic or skin cells of normal and patients with ALS, paving the way for opportunities to develop patient-specific treatments [91, 223]. Motoneurons derived from induced pluripotent stem cells (iPS cells) from ALS patients, harboring SOD1, C9orf72, and fused-in-sarcoma (FUS) mutations, have been reported to display reduced delayed-rectifier K^+^ current (Kv7 or M-current) amplitudes relative to control-derived motor neurons [91]. The M-current activator retigabine both blocks the hyperexcitability and improves motor neuron survival* in vitro* when tested in SOD1 mutant ALS patient iPS cell-derived motoneurons in culture [91]. A more recent study also reported initial hyperexcitability followed by progressive loss of action potential output and synaptic activity, due to a progressive decrease in voltage-activated Na^+^ and K^+^ currents, in patient iPS cell-derived motoneurons, harboring transactivation response element (TAR) DNA-binding protein (TARDBP) or C9ORF72 ALS-causing mutations [223]. These studies from human iPS cell-derived motoneurons are consistent with our results, discussion, and conclusions primarily based on mice models of ALS.

## 6. Potential Roles of Microglia in Motoneuron Plasticity and Neurodegeneration

It is increasingly accepted that ALS is a complex neurodegenerative syndrome that involves not only motoneurons but also a wide range of different tissues and cell types, including interneurons, muscle cells, astrocytes, oligodendrocytes, and microglia [178, 193, 194, 214, 230, 240, 241]. Although ALS primarily affects motoneurons, astrocyte and microglia activation and skeletal muscle atrophy (sarcopenia) are also typical hallmarks of the disease. However, the functional relationship between motoneurons, astrocytes, microglia, and skeletal muscle in the pathogenic process remains unclear. Neuroinflammation is evident in rodent models of inherited ALS overexpressing mutant SOD1 and in ALS human patients [176, 240–246]. A consistent neuropathologic feature of ALS is the extensive inflammation around motor neurons evidenced by the accumulation of reactive astrocytes and activated microglia [240–244].

**Figure 9:**
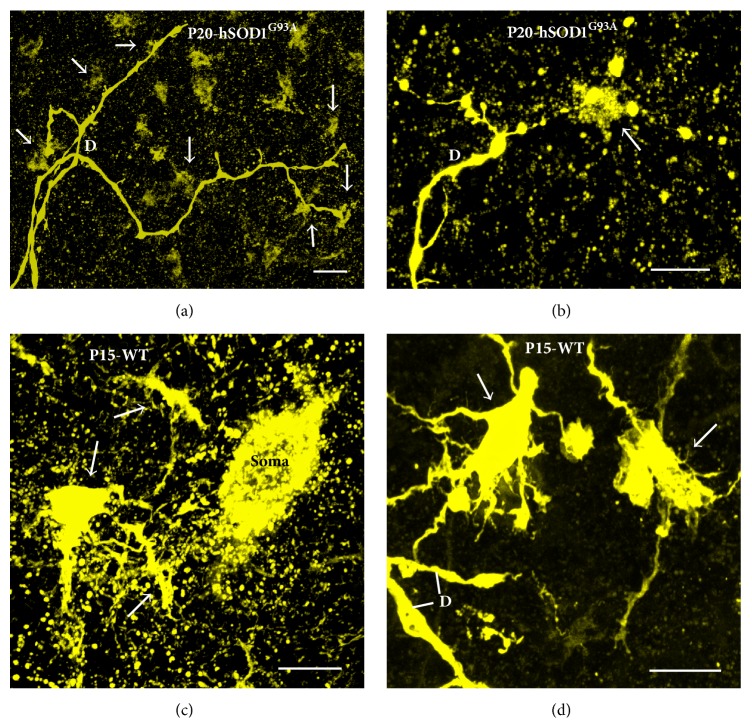
Microglial control of motoneuronal territory. Microglia were indirectly labeled by Neurobiotin, which they engulfed by phagocytosis from Neurobiotin-filled motoneurons, evident from detection of Neurobiotin both in dye-filled motoneuron and microglia. Neurobiotin was visualized by incubating brain slices in Streptavidin Cy3, as usual (standard) in all our preparations presented in this study (for details see Kanjhan and Vaney, 2008 [25], and Kanjhan and Bellingham, 2013 [26]). (a) A mosaic of activated microglia (arrows; with amoeboid appearance and loss of stellate processes) seen in close contact to the Neurobiotin-filled distal dendrites of a hypoglossal motoneuron in a brain slice from a P20 mice overexpressing the mutated human SOD1^G93A^ gene. (b) An example of resident microglia (arrow) engulfing distal dendrites (D) of a dye-filled hypoglossal motoneuron from hSOD1^G93A^ mutant mouse, as indicated by the blebbing of the dendrite and that both dendrite and microglia contain Neurobiotin. (c-d) Resident microglia (arrows), activated by blocking inhibitory synaptic transmission by addition of 5 *μ*M bicuculline and 2 *μ*M strychnine for >15 minutes, engulfing the soma (c) and dendrites (d) of a dye-filled hypoglossal motoneuron in brainstem slices from P15 WT mice. Bright appearance of microglia is due to Neurobiotin taken by phagocytosis from the soma and dendrites of motoneuron. Note swelling, beading, blebbing, and vacuolization in soma (c), dendrites (b, d), and spines (b). Scale bars = 10 *μ*m.

Microglia are the resident macrophages in the nervous system where they form a nonoverlapping mosaic or microglial network, which monitors and controls the environment and activity of neurons (Figure 9(a)) [247–249]. Microglia are considered the most susceptible sensors of neuronal environment and brain pathology and have additional roles in providing cytokines, growth factors, and neurotransmitters during development and neuronal plasticity [247–249]. Microglia are located in close proximity to synapses; with their highly dynamic and motile processes containing actin-based cytoskeleton, they can scan their territorial domains and display transient interactions with the synapses [247–249]. Microglial surveillance and synaptic pruning have been shown to be important in normal brain development and synaptic maturation [248, 250, 251]. Therefore deficits or changes in microglial function may contribute to synaptic abnormalities seen in neurodegenerative and neurodevelopmental diseases [246, 248, 250–252]. Signs of nervous tissue damage, lesion, or dysfunction result in a complex and multistage activation process that converts resident microglial cells to their activated form [247, 249]. Once activated, microglia can migrate to the injured or dysfunctional site, proliferate, and form new processes; and then they are able to destroy neurons either by direct phagocytosis or by indirectly secreting neurotoxic substances [245, 247, 249]. Selective changes in motoneuronal activity, such as synaptic hyperactivity in hSOD1^G93A^ mutants (Figure 7(b)), or pharmacologic block of the inhibitory synaptic transmission leading to disinhibited motoneuron bursting [184, 217] will increase metabolic and energy demand by depleting intracellular ATP (see above) and this will subsequently activate microglia to attack and phagocytose the motoneuronal soma and dendrites (Figure 9). Microglial attack and phagocytosis were seen in presymptomatic hSOD1^G93A^ mutant mice in a minority (~5%) of cells dye-filled with Neurobiotin (Figures 9(a)-9(b)). By contrast, microglial attack and phagocytosis were never observed in WT hypoglossal motoneurons under normal conditions. However, pharmacological blocking of inhibitory synaptic transmission activated microglia acutely (within minutes) and the microglial response was intense and seen in all the cells tested (*n* = 6) from the hSOD1^G93A^ mutant or WT mice (Figures 9(c)-9(d)). Therefore it is likely that the intensity of microglial response correlates with the level of motoneuronal activity and metabolic demand due to intracellular ATP depletion. Although our example shown here is acute, as blocking inhibitory synaptic transmission occurs within minutes (Figures 9(c)-9(d)), it is possible that lower levels of chronically increased activity may also cause significant damage over time [240, 242–244]. Once microglia are activated, we see swelling and vacuolization in soma, dendrites, filopodia, and spines of motoneurons (Figure 9), subsequently resulting in rapid disintegration of affected motoneurons. In support of this idea, a recent study has shown that the modulation of microglial activation by Fasudil, a Rho kinase inhibitor drug, prolongs survival and improves motor function in hSOD1^G93A^ mice [253].

## 7. Conclusions

Previous studies and our observations suggest that filopodia and dendritic spines are central structural elements of motoneuronal development and plasticity under both normal and neuropathological conditions. Both lumbar and hypoglossal motoneurons display dense filopodia and spine-like structures in their somatodendritic domains at late embryonic (prenatal) and newborn stages. During normal postnatal maturation of motoneurons, the density of filopodia reduced whilst spine-like stubby processes increased until around P15 and then decreased by P30. Spine distribution shifted towards the distal dendrites, and spine density decreased and spines became shorter and thick (stubby; 0.1 to 0.4 *μ*m long) with postnatal maturation. This coincided with a decreased frequency and increased amplitude of excitatory postsynaptic currents in motoneurons by ~2- to 3-fold at P30. Our findings, consistent with Vaughn's synaptotropic hypothesis, suggest that filopodia may be involved in spinogenesis and synaptogenesis, as well as dendritic growth and branching critical for circuit formation and synaptic plasticity during embryonic/prenatal and neonatal development. The sequences of spinogenesis/synaptogenesis in motoneurons differ from pyramidal and Purkinje cells and fit with a unique unified-hybrid model (Figure 8). Dendritic length and branching and filopodia and spine density, shape, and length are all dynamic and regulated by development (e.g., genetics) and by neuronal activity determined by synaptic or intrinsic properties. The soma and dendritic trees of motoneurons receive highly orchestrated excitatory and inhibitory synaptic inputs, allowing motoneurons to control and coordinate highly complex and refined motor tasks. Any significant and prolonged changes to the balance of excitatory-inhibitory synaptic inputs can result in synaptic hyperactivity and changes in intrinsic membrane properties (i.e., hyperexcitability), with associated changes in neuronal dendritic tree, filopodia, and spine morphology. Remodeling of synaptic, intrinsic membrane, and morphological properties of motoneurons can ultimately lead to excitotoxicity and subsequent motoneuronal damage. Microglial synaptic pruning and phagocytosis may shape this remodeling process. Future studies need to address the molecular mechanisms driving the changes in microglia and motoneurons during normal development, in the genesis of synaptic hyperactivity, and in subsequent motoneuron loss in neurodegenerative and neurodevelopmental diseases. Finally, we are hoping that this contribution will make an impact and stimulate new research on dendritic spine structure and function during development and disease, particularly in the motoneuron field.

## Abbreviations

ADF/cofilin: Actin depolymerizing factor/cofilin
Akt: Protein kinase B
ALS: Amyotrophic lateral sclerosis
AMPA: *α*-Amino-3-hydroxy-5-methyl-4-isoxazolepropionic acid
Arp2/3: Actin-related proteins 2/3
ATP: Adenosine triphosphate
BDNF: Brain-derived neurotrophic factor
bFGFs: Basic fibroblast growth factors
C9ORF72: Chromosome 9 open reading frame 72
CaMKII: Calcium/calmodulin-dependent protein kinase II
cAMP: Cyclic adenosine monophosphate
cdc42: Cell division cycle 42
CT: Cholera toxin
Dscam1: Down's syndrome cell adhesion molecule 1
DNA: Deoxyribonucleic acid
E: Embryonic day
ECM: Extracellular matrix
EGF: Epithelial growth factor
EPSCs: Excitatory postsynaptic currents
EPSPs: Excitatory postsynaptic potentials
Ena/Vasp: Enabled vasodilator-stimulated phosphoprotein
ERM: Ezrin-radixin-moesin
Err3: Estrogen receptor-related protein 3
GABA: Gamma-aminobutyric acid
F-actin: Filamentous actin
FF: Fast-twitch fatigable
FR: Fast-twitch fatigue resistant
Fus: Fused in sarcoma
HRP: Horse radish peroxidase
hSOD1: Human Cu/Zn-superoxide dismutase
G-actin: Globular actin
GAD-67: 67 kDa glutamic acid decarboxylase
GFP: Green fluorescent protein
GluR1: AMPA receptor type 1
GluR2: AMPA receptor type 2
GTPases: Guanosine triphosphatases
iPS cells: Induced pluripotent stem cells
IPSCs: Inhibitory postsynaptic currents
IPSPs: Inhibitory postsynaptic potentials
Kif5: Kinesin-1 family 5
KO: Knockout
Kv2.1: Voltage-dependent delayed rectifier K^+^ channel 2.1
LMC: Lateral motor column
MAPK: Mitogen-activated protein kinase
mGluR: Metabotropic glutamate receptor
Miro1: Mitochondrial Rho1
MMC: Medial motor column
MW: Molecular weight
Myo10: Myosin-X
MyTH4-FERM: Myosin tail homology 4-for protein 4.1, ezrin, radixin, and moesin
NADPH: Nicotinamide adenine dinucleotide phosphate-oxidase
NCAM: Neuronal cell adhesion molecules
NeuN: Neuronal nuclear antigen
NMDA: * N*-Methyl-D-aspartic acid
NMJ: Neuromuscular junction
NOX: NADPH-dependent membrane oxidase
NR3B: NMDA receptor type 3B
P: Postnatal day
PAK1: P21-activated kinase 1
PFN-1: Profilin 1
PI3K: Phosphatidylinositol 3-kinase
PKC: Protein kinase C
PLC: Phospholipase C
PLS-3: Plastin 3
PSD-95: Postsynaptic density 95
Rac1: Ras-related C3 botulinum toxin substrate 1
Ras: Rat sarcoma
Ras/ERK: Rat sarcoma/extracellular signal regulated kinases
Rho: Ras homolog
ROS: Reactive oxygen species
S: Slow-twitch
SK3: Small conductance Ca^2+^-activated K^+^ channel subtype 3
SMA: Spinal muscular atrophy
*smn 1*: Survival motor neuron 1 gene
SKN: Serum inducible kinase
srv2: Suppressor of *ras* ^*Val*14^
TARDBP: Transactivation response element (TAR) DNA-binding protein
TDP-43: Transactivation response element (TAR) DNA-binding protein-43
TM-agrin: Transmembrane agrin
TnC: Tenascin C
TrkB: Tyrosine kinase B (also called, tropomyosin-related kinase B)
VPS9: Vacuolar protein sorting-associated protein 9
VARP: VPS9-ankyrin repeat protein
VGAT: Vesicular inhibitory amino acid transporter
VGLUT2: Vesicular glutamate transporter 2
WASP: Wiskott-Aldrich syndrome protein
WGA: Wheat-germ agglutinin
Wnt: Wingless/Int-1
WT: Wild type
YFP: Yellow fluorescent protein.
